# Hydrogen Bonding in Natural and Unnatural Base Pairs—A Local Vibrational Mode Study

**DOI:** 10.3390/molecules26082268

**Published:** 2021-04-14

**Authors:** Nassim Beiranvand, Marek Freindorf, Elfi Kraka

**Affiliations:** Computational and Theoretical Chemistry Group (CATCO), Department of Chemistry, Southern Methodist University, 3215 Daniel Ave, Dallas, TX 75275-0314, USA; nbeiranvand@smu.edu (N.B.); mfreindorf@smu.edu (M.F.)

**Keywords:** natural base pairs, unnatural base pairs, hydrogen bonding, vibrational spectroscopy, local vibrational mode analysis

## Abstract

In this work hydrogen bonding in a diverse set of 36 unnatural and the three natural Watson Crick base pairs adenine (A)–thymine (T), adenine (A)–uracil (U) and guanine (G)–cytosine (C) was assessed utilizing local vibrational force constants derived from the local mode analysis, originally introduced by Konkoli and Cremer as a unique bond strength measure based on vibrational spectroscopy. The local mode analysis was complemented by the topological analysis of the electronic density and the natural bond orbital analysis. The most interesting findings of our study are that (i) hydrogen bonding in Watson Crick base pairs is not exceptionally strong and (ii) the N–H⋯N is the most favorable hydrogen bond in both unnatural and natural base pairs while O–H⋯N/O bonds are the less favorable in unnatural base pairs and not found at all in natural base pairs. In addition, the important role of non-classical C–H⋯N/O bonds for the stabilization of base pairs was revealed, especially the role of C–H⋯O bonds in Watson Crick base pairs. Hydrogen bonding in Watson Crick base pairs modeled in the DNA via a QM/MM approach showed that the DNA environment increases the strength of the central N–H⋯N bond and the C–H⋯O bonds, and at the same time decreases the strength of the N–H⋯O bond. However, the general trends observed in the gas phase calculations remain unchanged. The new methodology presented and tested in this work provides the bioengineering community with an efficient design tool to assess and predict the type and strength of hydrogen bonding in artificial base pairs.

## 1. Introduction

Deoxyribonucleic acid (DNA) is one of the most intriguing biomolecules found in nature; it basically encodes all necessary information for the diverse functions of life [1,2,3]. In the early 1950s, a race started to determine the structure of this fascinating biomacromolecule [4,5,6,7]. Linus Pauling and Robert B. Corey published an article in February of 1953 [8] proposing a triple helix DNA structure with the bases oriented at the outside. Although Pauling’s and Corey’s model was proven to be incorrect by Watson and Crick a couple of months later [9] they were one of the first scientists who came to the important conclusion that genes are segments of DNA that contain the code for a specific protein determining its function in different cells in the body, which can be considered as a first milestone for gene sequencing and gene cloning [10,11]. As described by Watson and Crick in their landmark paper [9], DNA forms a double strand helix, in which the four nucleobases guanine, cytosine, adenine and thymine of the DNA single strands form two pairs, guanine-cytosine (GC) and adenine-thymine (AT), also known as Watson-Crick or natural base pairs (**NBP${}_{s}$**), bound to each other by intermolecular hydrogen bonds [12,13,14]. Why nature has decided to use just these two base pair combinations remains one of the greatest mysteries [15,16].

An increasing number of efforts have been made to use Nature’s genius DNA concept in practical applications. For example, the physical and chemical properties of DNA have been exploited to create machines that are both encoded by and built from DNA molecules [17,18]. Utilizing DNA as a material building block in molecular and structural engineering has already led to the creation of numerous molecular-assembly systems and materials at the nanoscale [19]. Substantial efforts have been made to expand the genetic alphabet of DNA by introducing other base pair combinations, so-called unnatural base pairs (**UBP${}_{s}$**) to increase nucleic acid functionalities [20,21,22,23,24,25,26,27,28,29,30,31]. Recently, modifications of DNA containing four **NBP${}_{s}$** and four additional **UBP${}_{s}$** which efficiently replicated [32,32] were reported. A range of **UBP${}_{s}$**, termed xeno nucleic acids (**XNA${}_{s}$**) were introduced [33,34]. **XNA${}_{s}$** are constructed by replacing natural bases, sugars, and phosphate linkages of DNA with artificial structures in order to synthesize potential alternative genetic materials, which may open new horizons of genetically modified organisms [18,35,36,37,38,39].

**Figure 1:**
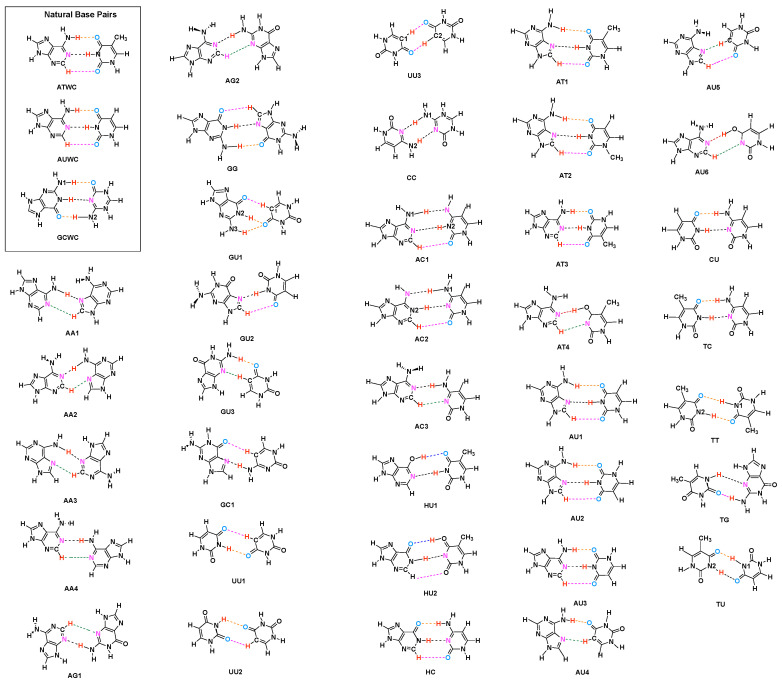
Investigated Base Pairs. (Key: A = Adenine, T = Thymine, C = Cytosine, G = Guanine, U = Uracil, H = Hypoxanthine. Different HBs are indicated by different color: N–H⋯N = Black, N–H⋯O = Orange, C–H⋯O = Pink, C–H⋯N = Green, O–H⋯O = Blue, O–H⋯N = Red). The selection of **UBP${}_{s}$** is described in the text.

A key feature of the base pairs is their link via hydrogen bonds (HB) [40,41,42,43,44,45]. Therefore, a comprehensive study of the hydrogen bonds formed between the base pairs is imperative (i) for the deeper understanding of the structure and biological function of DNA, and (ii) to assess the qualification of designer **UBP${}_{s}$**. HBs are one of the most important interactions found in biochemical molecules. Already in the 1950s Linus Pauling explored together with Robert B. Corey the importance of hydrogen bonding in proteins [46,47,48], work which contributed to his Nobel Price in Chemistry, awarded to him in 1954 for his research into the nature of the chemical bond and its application to the elucidation of the structure of complex substances [49,50]. Up to now HBs have been the object of numerous experimental and theoretical investigations [45,51,52,53], and because of the complex interplay between different components, their nature is still subject of an ongoing debate [41,42,54]. Using intermolecular HBs as the key feature of base pairs selectivity GC base pairs with different bonding patterns and atomic organization were suggested [55,56], as well as different **UBP${}_{s}$** estimating the HBs via calculated interaction energies [57]. Brovarets and co-workers [58] discussed the formation of C–H⋯O/N bonds in **UBP${}_{s}$** and showed that these HBs incorporate equally well into the structure of DNA. The effect of alkali metal cations on length and strength of HBs in DNA base pairs has been recently discussed [59] concluding that metal cations may help the base pairs stabilize to varying degrees depending on their position. DNA mutates as a result of proton transfer reactions. External electrostatic fields can modulate these reactions in DNA. Cerón-Carrasco and co-workers showed that mutagenic effects of high intensity electric fields on DNA have an impact on hydrogen bonding [60]. For example, during a pulse, HBs are elongated by widening the DNA strands, a reversible change once the electric field is removed. They also showed that in the guanine-cytosine base pair, the rate constants of proton transfer reactions can be changed by an electric field which is also in control of the mechanism of those reactions [61]. Through the design of a nanofluidic system that incorporates a number of synergistic functionalities displayed by both DNA molecules and the device itself, Kounovsky-Shafer and co-worker developed [62] an electrostatically inspired method for genome analysis. Molecular loading into nano-slits is aided by low ionic strength pressures, which are dynamically combined for efficient transport and temporal regulation. Proton transfer along DNA’s hydrogen bonds can lead to gene mutation and, possibly, cancer. Slocombe and co-workers investigated energy barriers and tunneling rates of hydrogen transfer of canonical and tautomeric Watson-Crick DNA base pairs [63]. They showed that the guanine-cytosine structure plays a role in spontaneous point mutations, if it survives long enough to pass via the RNA polymerase. However, they found a slightly reverse reaction barrier for adenine-thymine, suggesting that the adenine-thymine tautomer is unstable [63]. As a result, populating the tautomeric state through double proton transfer from the canonical state is unlikely a biologically important mechanism for spontaneous point mutations. The tautomeric state seems to be more likely to occur through proton tunneling in the hydrogen-bonded conformation than in the single-stranded conformation, according to combined studies of the hydrogen-bonded and dissociated forms of the DNA bases. Florián and Leszczyński [64] studied the energetic provisions for mutational DNA mechanisms. They showed that the guanine-cytosine base pair has more structural variability than previously thought. This base pair’s ion-pair and imino-keto/amino-enol forms are energetically available, though the probability of their formation is less than 10${}^{-6}$ while they are significantly nonplanar. While all these efforts have definitely increased our knowledge about DNA they lack one important ingredient, a quantitative measure of the intrinsic HB strength, which we introduce in this work. Based on the local vibrational mode analysis, originally introduced by Konkoli and Cremer [65,66,67,68,69] we assessed the intrinsic strength of the HBs of the **NBP${}_{s}$** of DNA and the adenine-uracil (AU) base pair found in RNA, and a set of 36 **UBP${}_{s}$**, shown in Figure 1 via local mode force constants, complemented with electron density and molecular orbital analyses. The **UBP${}_{s}$** where chosen from Brovarets’ set which was designed to span over a large variety of different **UBP${}_{s}$** with both classical and non-classical HBs [58], complemented with several **UBP${}_{s}$** purely connected by classical HBs, (CU, TC, TT, TG, TU; see Figure 1) [70]. The main focus of our work was to shed some new light into the question why Nature chose the **NBP${}_{s}$** from the perspective of hydrogen bonding, exploring if hydrogen bonding in **UBP${}_{s}$** differ substantially from that in **NBP${}_{s}$**. Based on our results we developed a roadmap for the design of **UBP${}_{s}$**. The paper is structured in the following way. In the methodology section methods used in this work are described and computational details are provided. The results and discussion presents our finding, and conclusion and an outlook are made in the final section.

## 2. Methodologies

In this section, first the tools applied in this work are introduced, i.e., the local vibrational mode analysis (LMA), originally introduced by Konkoli and Cremer [65,66,67,68,69] and the topological analysis of the electron density ρ(r) based on Bader’s quantum theory of atoms in molecules (QTAIM) [71,72]. Then the computational details are described.

### 2.1. The Local Vibrational Mode Analysis

A comprehensive mathematical derivation of the *local vibrational mode theory* can be found in a recent review article [73]. Therefore, in the following the most important essentials are summarized. The (3N-L) normal vibrational modes of a molecule being composed of N atoms (L = 5 for a linear and 6 for a nonlinear molecule) contain important information about the electronic structure and bonding. However, they tend to delocalize over the molecule due to the coupling of the atomic motions [74,75]. Therefore, one cannot directly derive an intrinsic bond strength measure from the normal modes. There are two coupling mechanisms, electronic coupling associated with the potential energy content of the vibrational mode and mass coupling associated with the kinetic energy. The electronic coupling between normal vibrational modes is promoted by the off-diagonal elements of the force constant matrix $F^{q}$ in internal coordinates **q** and it is eliminated by diagonalizing $F^{q}$, i.e., solving the fundamental equation of vibrational spectroscopy and transforming to normal mode coordinates [74,75]:

(1) $$F{\;}^{q}\;D\;=\;G^{-1}\;D\;\Lambda$$

where **G** represents the Wilson mass-matrix. Normal mode eigenvectors $d_{\mu }$ in internal coordinates **q** are collected in matrix **D**, and the corresponding vibrational eigenvalues $\lambda_{\mu }=4\pi^{2}c^{2}\omega_{\mu }$ in diagonal matrix Λ, where $\omega_{\mu }$ represents the harmonic vibrational frequency of mode $d_{\mu }$ in reciprocal cm, c is the speed of light, and μ=(1,2...N−L). Solving of Equation (1), e.g., diagonalizing the Wilson equation leads to the diagonal force constant matrix K in normal coordinates Q, which is free of electronic coupling Equation (2) [74,75]:

(2) $$K{\;}^{Q}\;\;=\;D^{†}F{\;}^{q}\;D$$

However, this procedure does not resolve the mass-coupling which often has been overlooked. Konkoli and Cremer [65,66,67,68,69] solved this problem by introducing a mass-decoupled equivalent to the Wilson equation to derive mass-decoupled local vibrational modes $a{}_{i}$ directly from normal vibrational modes $d_{i}$ and the K matrix via Equation (3):

(3) $$a_{i}\;=\;\frac{K^{-1}d_{i}^{†}}{d_{i}\;K^{-1}\;d_{i}^{†}}$$

For each local mode *i*, one can define a corresponding local model frequency $\omega_{i}^{a}$, a local force constant $k_{i}^{a}$, and a local mode mass $G_{i,i}^{a}$ [65]. The local mode frequency $\omega_{i}^{a}$ is defined by:

(4) $${\left(\omega_{i}^{a}\right)}^{2}\;=\;\frac{G_{i,i}^{a}\;k_{i}^{a}}{4\pi^{2}c^{2}}$$

and the corresponding local mode force constant $k_{i}^{a}$ by:

(5) $$k_{i}^{a}\;=\;a_{i}^{†}\;K\;a_{i}$$

Local vibrational modes have a number of unique properties. Zou, Kraka and Cremer [67,68] verified the uniqueness of the local vibrational modes via an adiabatic connection scheme between local and normal vibrational modes. In contrast to normal mode force constants, local mode force constants have the advantage of not being dependent of the choice of the coordinates used to describe the target molecule and in contrast to vibrational frequencies they are independent of the atomic masses. They are of high sensitivity to electronic structure differences (e.g., caused by changing a substituent) and directly reflect the intrinsic strength of a bond or weak chemical interaction as shown by Zou and Cremer [76]. Thus, local vibration stretching force constants have been utilized as a unique measure of the intrinsic strength of a chemical bond [69,77,78,79,80,81,82,83,84,85,86,87,88,89,90] or weak chemical interaction [91,92,93,94,95,96,97,98,99,100,101,102,103,104,105,106,107,108,109,110,111,112,113,114] based on vibration spectroscopy.

In this work, we used local stretching frequencies $\omega^{a}$ and stretching force constants $k^{a}$ for the characterization and comparison of the intrinsic strength of the intermolecular HBs of **NBP${}_{s}$** and their **UBP${}_{s}$** counter parts.

It is convenient to base the comparison of the bond strength of a series of molecules on a bond strength order (BSO) *n* rather than on a comparison for local force constant values. Both are connected via a power relationship according to the generalized Badger rule derived by Cremer and co-workers [79]:

(6) $$BSO\;n\;=\;a\;\left(k^{a}\right){\;}^{b}$$

The constants *a* and *b* in Equation (6) can be determined from two reference with known (BSO) *n* values and the requirement that for a zero force constant the BSO *n* is zero. For HBs, we generally use as references the FH bond in the FH molecule with BSO n=1 and the FH bond in the [H⋯F⋯H]${}^{-}$ anion with BSO n=0.5 [91,103,109]. For an ωB97X-D/6-31++G(d,p) model chemistry, applied in this study, this leads to $k^{a}$(FH) = 9.782 mdyn/Å, $k^{a}$(F⋯H) = 0.901 mdyn/Å, a=0.515 and b=0.291. According to Equation (6) the OH bond in H_2_O has a BSO *n* value of = 0.966. We scaled the reference values [109], so that the BSO *n* of the OH bond in H_2_O is 1.

### 2.2. QTAIM and NBO Analysis

The Quantum Theory of Atoms-In-Molecules developed by Bader [115,116] presents a theoretical scheme for identifying, analyzing and characterizing chemical bonds and interactions via the topological features of the total electron density ρ(r). In this work we used QTAIM as a complementary tool to the local mode analysis to determine the covalent/electrostatic character of internal HBs via the Cremer-Kraka criterion [117,118,119] of covalent bonding.

The Cremer-Kraka criterion is composed of two conditions; necessary condition: (i) existence of a bond path and bond critical point $r_{c}$ = *c* between the two atoms under consideration; (ii) sufficient condition: the energy density $H(r_{c})$ = $H_{c}$ is smaller than zero. H(r) is defined as:

(7) H(r)=G(r)+V(r)

where G(r) is the kinetic energy density and V(r) is the potential energy density. A negative V(r) corresponds to a stabilizing accumulation of density whereas the positive G(r) corresponds to depletion of electron density [118]. As a result, the sign of $H_{c}$ indicates which term is dominant [119]. If $H_{c}<0$, the interaction is considered covalent in nature, whereas $H_{c}>0$ is indicative of electrostatic interactions.

In addition to the QTAIM analysis we used the Natural Bond Orbital (NBO) population analysis of Weinhold and co-workers [120,121,122] in order to obtain atomic charges and the charge transfer between the two monomers forming the base pair.

### 2.3. Computational Methods

Geometry optimizations and harmonic frequency calculations were performed with the Gaussian 16 program [123] using the ωB97X-D functional [124,125] in combination with Pople’s 6-31++G(d,p) basis set [126,127,128,129]. An ultra-fine grid was used for the numerical DFT integration [130]. All local mode analysis calculations were carried out with the program package LModeA [73,131]. The NBO calculations were carried out with NBO 6 [122]. The QTAIM analysis was performed with the AIM2000 [132] software for calculating the bonds critical points and visualizing the bonds path. Binding energies (BE) were calculated also at the ωB97X-D/6-31++G(d,p) level of theory, where the counterpoise correction of Boys and Bernardi [133] was used to correct for basis set superposition errors.

In order to assess the validity of our gas phase study we also analyzed the intrinsic HB strength for four Watson–Crick base pairs, two **AT**, and two **GC** in a DNA environment. The analysis was based on combined Quantum mechanics/Molecular mechanic (QM/MM) calculations [134], which were performed for each base pair using initial DNA coordinates from an X-ray structure of a synthetically constructed DNA dodecamer, PDB entry 6CQ3 [135]. The QM/MM geometry optimization were performed using ONIOM [136] with electronic embedding without constraints, followed by vibrational frequency calculations utilizing the ωB97X-D/6-31++G(d,p)/AMBER level of theory. For comparison we also calculated optimal geometries and vibrational frequencies of the **AT** base pair (labeled as **AT3${}_{gas}$**) and the **GC** base pair (labeled as **GC3${}_{gas}$**) in the gas phase, based on a starting geometries of the corresponding QM part of the QM/MM calculations at the ωB97X-D/6-31++G(d,p) level of theory. Calculations were performed with Gaussian16 [123]. Further details are given in the supporting information.

## 3. Results and Discussion

### 3.1. Internal HB Strength

**Figure 2:**
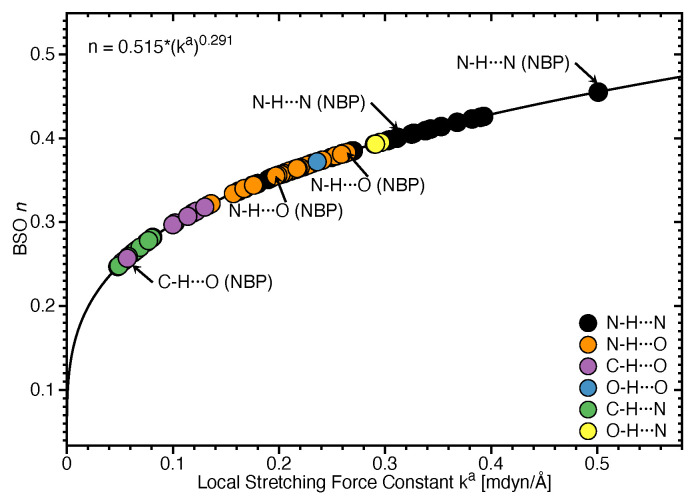
Bond strength order BSO *n* of the HBs for the **NBP${}_{s}$** and **UBP${}_{s}$** as a function of the corresponding local stretching force constant $k^{a}$ determined via Equation (6). Calculated at the ωB97X-D/6-31++G(d,p) level of theory.

Figure 2 shows the bond strength order BSO *n* of the HBs for the **NBP${}_{s}$** and **UBP${}_{s}$** as a function of the corresponding local stretching force constant $k^{a}$ derived from Equation (6). The obtained BSO *n* values for **NBP${}_{s}$** range from 0.256 to 0.455 while BSO *n* values for **UBP${}_{s}$** range from 0.247 to 0.426, which leads to the important observation that the HBs of the **UBP${}_{s}$** and **NBP${}_{s}$** investigated in this work fall in to the same range, or in other words, the HB strength of **NBP${}_{s}$** does not stand out in any particular way. The N–H⋯N bond shows the strongest BSO *n* values of either **NBP${}_{s}$** or **UBP${}_{s}$**, and the weakest HB in both **NBP${}_{s}$** and **UBP${}_{s}$** is the C–H⋯O bond. Also, **NBP${}_{s}$** are stabilized by three HBs while the majority of the **UBP${}_{s}$** are stabilized by two HBs. We found N–H⋯N, N–H⋯O and C–H⋯O in **ATWC**, **AUWC** and **GCWC** are joined by one N–H⋯N and two N–H⋯O, but in case of **UBP${}_{s}$** in addition of these HBs, O–H⋯O, C–H⋯N and O–H⋯N bonds were found. Different combinations of hydrogen donor and acceptor atoms appear to be the main difference between natural base pairs and unnatural base pairs (see Table S1 in the supporting information). In the following each individual HB type is discussed in more detail.

**Figure 3:**
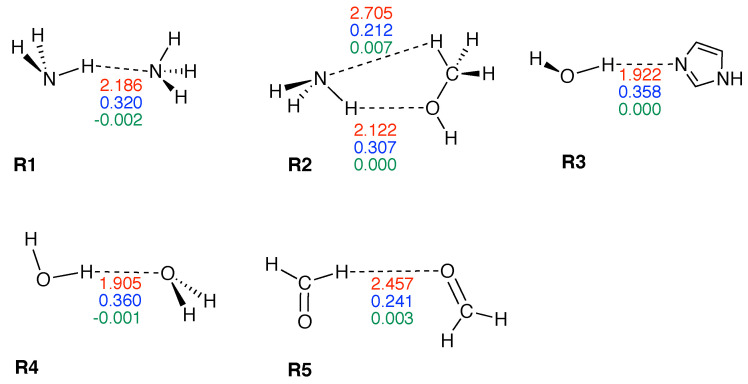
Reference complexes **R1**–**R5**. The intramolecular HB distance (Å) is given in red color, the corresponding BSO *n* value in blue color, and energy density H(c) (Hartree/Å${}^{3}$) in green color. Calculated at the ωB97X-D/6-31++G(d,p) level of theory.

**Figure 4:**
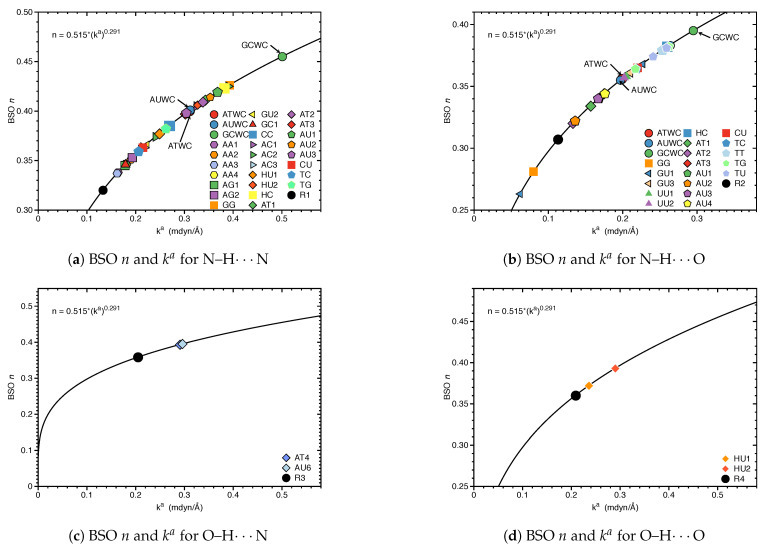
Bond strength order BSO *n* of the HBs for the **NBP${}_{s}$** and **UBP${}_{s}$** as a function of the corresponding local stretching force constant $k^{a}$ as determined via Equation (6). For comparison the HBs of the reference complexes are included. Calculated at the ωB97X-D/6-31++G(d,p) level of theory.

Figure 3 shows compounds **R1**–**R5** used to compare HBs properties in base pairs and representative reference molecules. In Figure 4a, we compare BSO *n* values and force constant $k^{a}$ for N–H⋯N bonds in base pairs and reference molecules. N–H⋯N bonds were found in 28 base pairs qualifying the N–H⋯N bond as the most favorable HB. BSO *n* values for N–H⋯N bonds range from 0.337 to 0.455, with **UBP${}_{s}$** values ranging from 0.337 to 0.426. It is interesting to note that all base pair N–H⋯N bonds are stronger than the HB bond of the reference molecule **R1** (BSO *n* = 0.320, see Table 1). The strongest N–H⋯N bond was found for **GCWC** which is stronger than the same bond in other **NBP${}_{s}$** (0.400 and 0.401 in **ATWC** and **AUWC**, respectively). The weakest N–H⋯N bond was found for **AA3**. Two gaps were observed in the Figure 4a; none of the **NBP${}_{s}$** and **UBP${}_{s}$** has a N–H⋯N bond in the BSO *n* range between 0.385 and 0.397 and between 0.426 and 0.455. One of the **UBP${}_{s}$** with the strongest N–H⋯N bonds, the **GG** base pair with a BSO *n* value of 0.426 has been discussed in so-called mismatched DNA causing genetic diseases [137,138]. The **GG** base pair has also the capability to form a H-bonding pattern close to that found in **NBP${}_{s}$**, i.e., being stabilized by three HBs, N–H⋯N, N–H⋯O and C–H⋯O (see Figure 2) making this pair an interesting candidate for xenobiology [33,34,139].

In Figure 4b, we compared BSO *n* values and force constant $k^{a}$ for N–H⋯O bond in base pairs and reference molecules. We found this HB in 21 base pairs. The BSO *n* values for N–H⋯O bonds range from 0.263 to 0.395. With the exception of **GG** (BSO *n* = 0.281) and **GU1** (BSO *n* = 0.263), all base pairs have stronger N–H⋯O bonds than reference molecule **R2** (BSO *n* value of 0.307, see Table 1). It should be noted that **GU1** makes two N–H⋯O bonds, two nitrogen atoms belong to guanine are donating electrons to an oxygen atom of uracil. The N–H⋯O bond in the center of this base pair has a BSO *n* = 0.368 which is stronger than the same bond in **R2**. The strongest N–H⋯O bond was found for **GCWC**, BSO *n* = 0.395 which is stronger than the same bond in other **NBP${}_{s}$** (0.356 and 0.355 in **ATWC** and **AUWC**, respectively). We observed three gaps in the Figure 4b, for BSO *n* between 0.322 and 0.334, between 0.344 and 0.355 and between 0.383 and 0.395. As the discussion above attests **GCWC** has the strongest HBs between the **NBP${}_{s}$** and **UBP${}_{s}$** and central N–H⋯N bond is stronger than N–H⋯O bond.

Figure 4c,d displays the BSO *n* values and force constant $k^{a}$ for O–H⋯N and O–H⋯O bonds. These HBs are less favorable and occur only in **UBP${}_{s}$**. The O–H⋯N bond was found in **AT4** and **AU6**, BSO *n* = 0.393 and 0.395, respectively, i.e., these HBs are stronger than the reference molecule **R3** (BSO *n* value of 0.358, see Table 1). The O–H⋯O bond was found in **HU1** and **HU2** with BSO *n* 0.372 and 0.393, respectively, i.e., both HBs are even stronger than the HB in the water dimer (BSO *n* = 0.360, see reference molecule **R4** in Table 1). The O–H⋯O bond in **HU2** is stronger than in **HU1**. The presence of a weak HB like C–H⋯O bond and a strong central N–H⋯N bond along with an O–H⋯O bond in **HU2** obviously increases the overall stability of this base pair.

Whereas base pair **HU1** is stabilized by O–H⋯O and N–H⋯N bonds, **HU2** exhibits three H-bond (O–H⋯O, N–H⋯N bonds and C–H⋯O). The C–H⋯O bond in **HU2** obviously makes a difference. Another weak HB, a C–H⋯N bond along with a O–H⋯N bond was found in **AT4** and **AU6** which appears to be stronger than the C–H⋯O bond. In summary, weak HBs play an important role for the stabilization of base pairs, which will be discussed in more detail in the following section.

### 3.2. Significance of Non-Classical HBs

A classical HB is defined as the interaction between a hydrogen atom bonded to a highly electronegative atom such as oxygen, nitrogen and fluorine and the lone pair of another such atom nearby [140,141]. Carbon is generally not considered as an electron donor which has led to this narrow definition of hydrogen bonding. In 2011, a new definition was introduced by the International Union of Pure and Applied Chemistry (IUPAC) [142,143], that emphasized the hydrogen donor does not always need to be one of the most electronegative atoms (oxygen, nitrogen and fluorine). One atom with a higher electronegativity than hydrogen is sufficient (non-classical HB). According to this new definition many interactions including less electronegative atoms such as carbon, chlorine, sulfur, phosphorus to act as the proton donor have been considered as HBs. In particular non-classical HBs were found to play a critical role for the structure and stability of biological systems, including DNA [144,145]. C–H⋯O bonding between phosphate groups and nitrogenous bases was identified as a stabilizing part in DNA stability.

**Figure 5:**
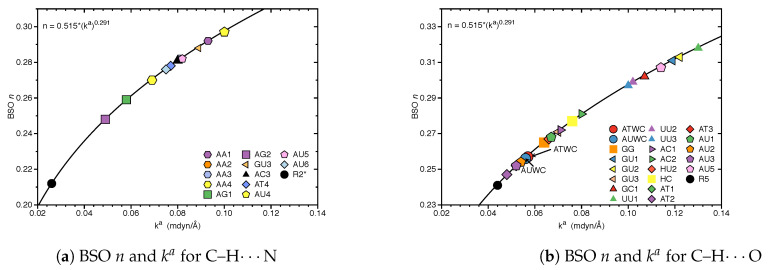
Bond strength order BSO *n* of the HBs for the **NBP${}_{s}$** and **UBP${}_{s}$** as a function of the corresponding local stretching force constant $k^{a}$ as determined via Equation (6). Calculated at the ωB97X-D/6-31++G(d,p) level of theory. For comparison the HB in the reference complexes are included.

In the case of **AT** and **AU**, the absence of a C–H⋯O bond causes their instability because the remaining oxygen in the minor groove can not be fully utilized as HB acceptor translating into a whole DNA structure [146]. Results presented in Figure 5 reveal the important stabilizing role of non classical HBs. In Figure 5a, the BSO *n* values and force constant $k^{a}$ for the C–H⋯N bond in base pairs are compared. This non-classical HB was found in 12 base pairs and it is less favorable for base pairs specially, we did not find it in any of the **NBP${}_{s}$**. BSO *n* values for C–H⋯N bond ranges from 0.248 to 0.297 which is much stronger than this HB in the reference molecule R2*(BSO *n* value of 0.212, see Table 1). The C–H⋯N bonds belong to the **AU6** and **AT4** with BSO *n* values 0.276 and 0.278, respectively, and are located in the middle of the Figure 5a. Thus they are strong enough to make the base pairs stable. In Figure 5b, the BSO *n* values and force constant $k^{a}$ for C–H⋯O bond in base pairs are compared. The non-classical C–H⋯O bond is much more favorable than C–H⋯N bond and it was found in 21 base pairs including both **NBP${}_{s}$** and **UBP${}_{s}$**. BSO *n* values range from 0.247 to 0.318, which is stronger than this HB in the reference molecule **R5** (BSO *n* value of 0.241, see Table 1). None of the base pairs shows a C–H⋯O bond in the gap between BSO *n* 0.281 to 0.297. This HB is naturally favored and it was found in **ATWC** and **AUWC** with BSO *n* value 0.257 and 0.256, receptively. However, we did not find any non-classical HBs in **GCWC**. **UU3** is stabilized just by two C–H⋯O bonds and **AU5** is formed by two non classical weak HBs (C–H⋯O bond and C–H⋯N bond). Our results show that the C–H⋯O bond is stronger than the C–H⋯N bond (where, the percentage of C–H⋯O bond with BSO *n* values ranges between 0.297 and 0.318 is 35.0%). This range is the most common HB type in water clusters [103]. In most cases, the base pairs are joined by three HBs, one of them is a weak non-classical C–H⋯O bond, which is more naturally favored and stronger. The C–H⋯N bonds were found always in **UBP${}_{s}$** which are stabilized by two HBs. We did not observe non classical HBs in 8 base pairs. These base pairs are stabilized by strong N–H⋯N bond from BSO *n* = 0.359 to BSO *n* = 0.455 and N–H⋯O bond from BSO *n* = 0.364 to BSO *n* = 0.395, and, C–H⋯O bond with BSO *n* = 0.372.

### 3.3. Covalent Character of HBs

**Figure 6:**
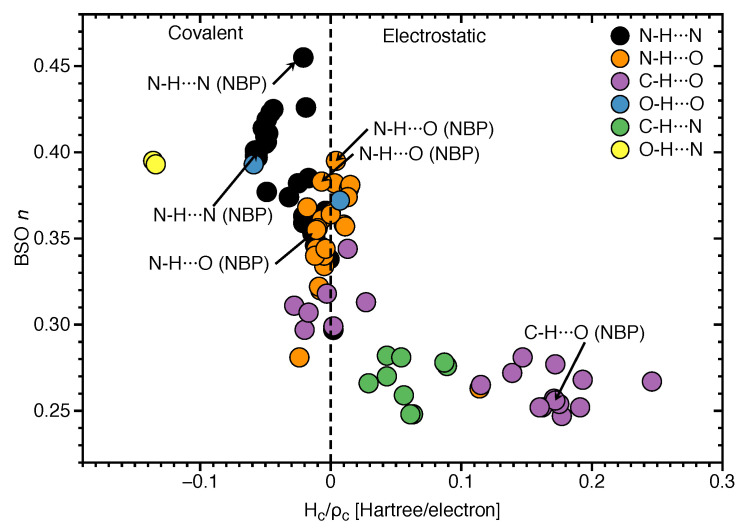
Correlation of BSO *n* and normalized energy density $H_{c}/\rho_{c}$. Calculated at the ωB97X-D/6-31++G(d,p) level of theory.

In the following we assess the covalent character of the **NBP** and **UBP** HBs via the normalized energy density $H_{c}/\rho_{c}$ for all of HBs investigated in this work. The electron density analysis is complemented with NBO charges of all atoms X–H⋯Y involved in hydrogen bonding, see Table S2 in the supporting information. In Figure 6, BSO *n* values are correlated with the corresponding $H_{c}/\rho_{c}$ values. In case of N–H⋯N bonds, $H_{c}/\rho_{c}$ values range from −0.058 Hartree/electron to 0.002 Hartree/electron. All of the base pairs have N–H⋯N bonds in the covalent region except the **GC1** with on the border value of $H_{c}/\rho_{c}$ = 0.002 Hartree/electron for N–H⋯N bond. Base pair **AU3** shows more covalent character (−0.058 Hartree/electron) of this HB compared to the same HB in other base pairs. According to our results $H_{c}/\rho_{c}$ for strongest N–H⋯N bond (belong to **GCWC**) is −0.021 Hartree/electron which is slightly less negative than the most covalent N–H⋯N (belonging to **AU3**) with $H_{c}/\rho_{c}$ = −0.058 Hartree/electron. However, most of the base pairs show that the increased strength of the N⋯H bond in N–H⋯N bond is correlated with a more covalent character of this bond (see Tables S1 and S2 in the supporting information).

To be more specific, the N⋯H bond of N–H⋯N belonging to **GC1** has the less covalent character ($k_{c}=0.002$ Hartree/electron) which shows the weakest BSO *n* = 0.297 for the same HB among all other base pairs. In Figure 6, the N⋯H bond of O–H⋯N shows the most covalent character ($H_{c}/\rho_{c}$ = −0.136 Hartree/electron in **AU6** and −0.134 Hartree/electron in **AT4**) between all types of HB that was investigated in this work. According the HB strength analysis, these are stronger HBs compared other HBs since, the strength of the HB also depends on the nature of donor (N, O, C), in addition of the electron density distribution of the lone pair of the HB acceptor atom (N, O). However, as we see in Figure 6, there are several central N⋯H bonds in the N–H⋯N bond with less covalent characters. This leads to the conclusion that if a base pair is formed with three HBs, a strong N–H⋯N bond is found in the middle. But the same HB in the base pair with two possible HBs is less covalent and weaker than the N⋯H bond in O–H⋯N. It should be noted that we didn’t found O–H⋯N bond in **WCBP${}_{s}$**, it means that the electrostatic interactions are more strongly felt in the interior non-polar environment of DNA where the bases form a pair. According Figure 6, C–H⋯N bonds are in the electrostatic region, but C–H⋯O bonds are speared in both covalent region with $H_{c}/\rho_{c}$ from −0.028 to −0.003 Hartree/electron and electrostatic regions with $H_{c}/\rho_{c}$ from 0.002 to 0.246 Hartree/electron. The C–H⋯O bonds belong to **ATWC** and **AUWC** are in the electrostatic region.

### 3.4. Intrinsic HB Strength and BEs

**Figure 7:**
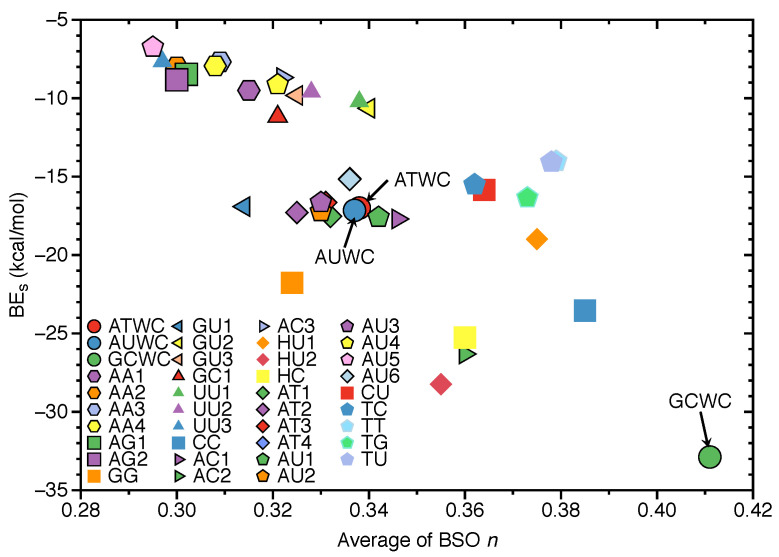
Correlation of the average of BSO *n* and binding energies (BE${}_{s}$). Calculated at the ωB97X-D/6-31++G(d,p) level of theory.

In Figure 7, the correlation between the average of BSO *n* of the HBs in each base pairs with BE${}_{s}$ is shown. The reason for using average BSO *n* is to account for the fact that the number of HBs differs in the base pairs, i.e., three or two HBs. There is some overall trend, i.e., stronger HBs are connected with larger BEs. However, the scattering of data points shows that there is no direct relationship between the two quantities, which is not surprising. The BE is a cumulative measure of the overall energy required to break a bond/weak interaction including the reorganization of the electron density and geometry relaxation of the dissociation product while the BSO *n* reflects intrinsic strength of the HB, as discussed above [101,102,105]. **GCWC** has the strongest BE (−32.88 kcal/mol) and the largest BSO *n* of 0.411 of all HBs investigated in this work. In contrast, the other two **NBP${}_{s}$** are found in the middle range. **AU5** has the weakest BE with −6.74 kcal/mol and the smallest average BSO *n* of 0.295.

### 3.5. HBs in the DNA Environment

**Figure 8:**
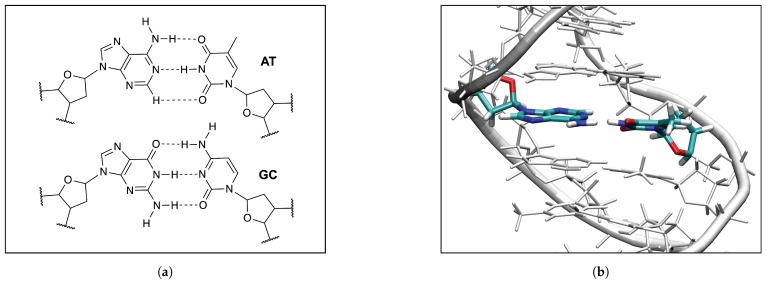
(**a**) The QM models of the **AT** and **GC** base pairs used in the QM/MM calculation; (**b**) The QM/MM optimized geometry of the **AT2${}_{DNA}$** base pair in DNA, the ωB97X-D/6-31++G(d,p) level of theory, the water molecules and the Na^+^ ions are not shown for clarity of the picture.

In order to evaluate the influence of the DNA environment on hydrogen bonding we compared the **AT** and **GC** base pairs in the gas phase (Figure 8a) and in the DNA (Figure 8b). The results are summarized in Table 2 and Table 3.

According to our calculations, the strongest HB of the **AT** base pairs in DNA, is observed for the N–H⋯N bonds, which are even stronger than in the **AT** base pair in the gas phase (0.383 and 0.420 mdyn/Å in **AT1** and **AT2** in DNA, and 0.318 mdyn/Å in **AT3** in the gas phase). However, the force constant of the O⋯H bond in the N–H⋯O bonds of the **AT** base pairs in DNA are smaller than the corresponding HBs in the gas phase (0.128 and 0.102 mdyn/Å in **AT1** and **AT2** in DNA, and 0.201 mdyn/Å in **AT3** in the gas phase), and the opposite trend is observed for the C–H⋯O non-classical HBs (0.156 and 0.141 mdyn/Å in **AT1** and **AT2** in DNA, and 0.060 mdyn/Å in **AT3** the gas phase). These results indicate that the DNA environment increases the strength of the central N–H⋯N HB and the C–H⋯O non-classical HB, and at the same time it decreases the strength of the N–H⋯O bond in the **AT** base pairs. As it is seen in Table 2 the increased strength of the N⋯H bond of the central N–H⋯N bond in DNA, is also correlated with the decreased strength of the N–H bond in this hydrogen bond (4.496 and 3.338 mdyn/Å in **AT1** and **AT2** in DNA, and 4.673 mdyn/Å in **AT3** the gas phase). A similar effect of the DNA environment is observed in our calculations of the **GC** base pairs. According to Table 2, the strongest hydrogen bond of the **GC** base pairs in DNA, is observed for the central N–H⋯N bond, similarly as in the **AT** base pairs, where the DNA environment increases the strength of the N⋯H bond in this hydrogen bond of the **GC** base pairs, and this increase is also correlated with a decrease of the N–H bond strength in this HB. Therefore, based on our QM/MM calculations of the two **AT** and two **GC** base pairs in DNA, we conclude that the DNA environment changes the electronic structure the central N–H⋯N bond of these base pairs, which makes the proton transfer between nitrogen atoms of the purine and pyrimidine bases easier. We can generally conclude that the gas phase calculations show the general features of HBs for the majority of the base pairs presented in this study. It has been confirmed in other studies that the Watson-Crick **AT** and **GC** base pairs are electronically complementary through proton transfer [147,148]. These results can be expanded to tautomeric base pairs where photoexcitation studies show a link between UV-excited DNA states and efficient charge production and transmission in DNA [147]. Base pair radical ions behave similarly to those created when ionizing radiation interacts with DNA [148,149,150,151]. Intermolecular hydrogen-bond distances in both tautomeric Watson-Crick base pairs are shorter than those in canonic base pairs. This means that after double-proton transfer in the canonic base pairs, the HBs become stronger [152,153,154].

NBO charges of atoms X–H⋯Y and energy densities of the X–H and the Y⋯H HBs of the **AT** and **GC** base pairs in gas phase and DNA are compared in Table 3. Because the central N–H⋯N bond is the strongest HB in the investigated base pairs, we focus in the following on a discussion of the NBO charges and $H_{c}$ for this particular HB. According to Table 3, the NBO atomic charges of the atoms involved in the N–H⋯N bond for the **AT** base pairs in DNA are similar to the NBO charges of these atoms based on the gas phase calculations (−0.605, 0.477, and −0.687 e in **AT1**; −0.606, 0.477, −0.685 e in **AT2**; −0.615, 0.477, −0.688 e in **AT3** for the N, H and N atoms, respectively; the first N atom belongs to the A base and the second N atom belongs to the T base). However, we observe changes in the $H_{c}$ for the N⋯H bond, when comparing the results from the DNA calculations and from the gas phase calculations (−0.0189 and −0.0661 Hartree/Å${}^{3}$ in **AT1** and **AT2** in DNA, and −0.0169 Hartree/Å${}^{3}$ in **AT3** in the gas phase). Similarly, there are also changes in the $H_{c}$ for the N–H bond (−3.0368 and −2.8613 Hartree/Å${}^{3}$ in **AT1** and **AT2** in DNA, and −3.0631 Hartree/Å${}^{3}$ in **AT3** in the gas phase). Therefore, the changes of the energy density at the bond critical point shown in Table 3 are consistent with the changes of the local mode force constants presented in Table 2, showing that the increased strength of the N⋯H bond in the DNA surrounding, is correlated with a more covalent character of this bond, and the decreased strength of the N–H bond in DNA is correlated with a less covalent character of this bond.

According to Table 3, the NBO atomic charges of the atoms involved in the N–H⋯N bond for the **GC** base pairs in DNA are similar to the NBO charges of these atoms based on the gas phase calculations (−0.658, 0.472, −0.655 e in **GC1**; −0.668, 0.472, −0.671 e in **GC2**; −0.677, 0.467, −0.661 e in **GC3** for the N, H and N atoms, respectively; the first N atom belongs to the G and the second N atom belongs to the C base). Similar to the **AT** base pairs, there are changes in the energy density at the bond critical point between the values obtained from the DNA calculations and from the gas phase calculations for the N⋯H bond (−0.0074 and −0.0067 Hartree/Å${}^{3}$ in **GC1** and **GC2** in DNA, and −0.0047 Hartree/Å${}^{3}$ in **GC3** in the gas phase), and for the N–H bond (−3.2082 and −3.1751 Hartree/Å${}^{3}$ in **GC1** and **GC2** in DNA, and −3.2291 Hartree/Å${}^{3}$ in **GC3** in the gas phase). Therefore, similar to the **AT** base pairs, the increased strength of the N⋯H bond in the **GC** base pairs in the DNA surrounding, is correlated with a more covalent character of this bond, and the decreased strength of the N–H bond in DNA is correlated with a less covalent character of this bond. The similar NBO atomic charges of the atoms involved in the N–H⋯N bond of the **AT** and **GC** base pairs in DNA and in the gas phase, confirm that the electrostatic interaction between these atoms is less important for the change of the strength in these bonds. Table 2 shows also a comparison of the QM/MM calculated in our study and the experimentally measured [135] distance between the hydrogen donor atom (X) and the hydrogen acceptor atom (Y) of the X–H⋯Y bond in the **AT** and **GC** base pairs in DNA. As a reference we also present in Table 2 this distance based on the QM gas phase calculations of the **AT** and **GC** base pairs. According to Table 2 the values of the calculated distance between the hydrogen donor and acceptor atoms are generally in the range the experimented values. Although this agreement is not perfect, a much better agreement with experiment is observed in our calculations of the **GC** rather than the **AT** base pairs, which can be explained by the smaller flexibility of the **GC** base pairs containing three classical HBs, in contrast to the **AT** base pairs having two classical and one non-classical HB.

## 4. Conclusions and Outlook

We investigated in this work intermolecular hydrogen bonding in a diverse set of 36 unnatural and the three natural Watson Crick base pairs adenine (A)–thymine (T), adenine (A)–uracil (U) and guanine (G)–cytosine (C). The hydrogen bond strength was assessed utilizing local vibrational force constants derived from the local mode analysis, originally introduced by Konkoli and Cremer as a unique bond strength measure based on vibrational spectroscopy. The local mode analysis was complemented by the topological analysis of the electronic density and the natural bond orbital analysis. Our study led to the following interesting insights:

- Hydrogen bonding in Watson Crick base pairs is not exceptionally strong and the N–H⋯N bond is the most favorable hydrogen bond in both unnatural and natural base pairs while O–H⋯N/O bonds are the less favorable in unnatural base pairs and not found at all in natural base pairs.
- In addition, non-classical C–H⋯N/O bonds play an important role for the stabilization of base pairs, especially C–H⋯O bonds in Watson Crick base pairs. This suggests that Nature’s choice to combine classical and non-classical hydrogen bonding should also be copied in the design of new unnatural base pair combinations.
- Hydrogen bonding in Watson Crick base pairs modeled in the DNA via a QM/MM approach showed that the DNA environment increases the strength of the central N–H⋯N bond and the C–H⋯O bonds, and at the same time decreases the strength of the N–H⋯O bond. However, the general trends observed in the gas phase calculations remain unchanged reflecting that electrostatic interactions with the environment are a less important factor determining the intermolecular hydrogen bond strength; an important validation of the gas phase model applied in this work.
- Natural base pairs do not possess larger binding energies than their unnatural counterparts. We also did not find a significant correlation between hydrogen bond strengths and binding energies, i.e., BSO *n* and BE values, as expected because these two quantities cannot directly be compared.
- We expect that the presence of base pairs with more nonclassical, i.e., weaker HBs in DNA will make the environment less covalent. During electron transfer these bonds will couple with specific vibrational modes of the DNA strand changing the electronic properties of the DNA. It has been documented [155,156,157,158] that these changes can stretch over 10 to 80 nucleobases accompanied by a decrease of the corresponding normal frequencies. When the DNA body lengthens, it becomes more mobile and less rigid. The experiment can only acquire normal vibrational frequencies of the backbone and the bases of DNA molecules characterized by coupled vibrational modes, while we can capture via LMA individual local frequencies from low to high and decode specific atomic motions, leading to more comprehensive and deeper insights into the stability of the DNA strand, which we will further explore in future work.
- The stability of the DNA double helix is mainly determined by (i) non-covalent interactions involving hydrogen bonds between A-T and G-C base pairs, (ii) stacking interactions between adjacent bases along the helix, and (iii) cross-interactions between base pairs [159]. Interactions outside the DNA double helix generally play a less important role [160]. The interplay between hydrogen bonding and stacking interactions in DNA has been the subject of several experimental [161,162,163,164,165] and theoretical investigations [159,166,167,168,169,170,171,172,173]. Based on DNA melting and energetics of the double helix [174], it has been recently suggested that in accordance with previous experiments [165,175] the stability of DNA double strands depends mainly on G-C base pair rich sequences. This is completely in line with our results identifying the hydrogen bonds of the G-C base pairs as one of the strongest. The local mode analysis can also quantitatively assess the strength of the stacking interactions between adjacent DNA bases along the helix, which is currently under investigation.

In summary, our study clearly reveals that not only the intermolecular hydrogen bond strength but also the combination of classical and non-classical hydrogen bonds play a significant role in natural base pairs, which should be copied in the design of new promising unnatural base pair candidates. Our local mode analysis, presented and tested in this work provides the bioengineering community with an efficient design tool to assess and predict the type and strength of hydrogen bonding in artificial base pairs.

## Figures and Tables

**Table 1:** Properties of hydrogen bonded reference complexes **R1**–**R5**.

Molecule	$k^{a}$	BSO *n*	R(HB)	ρ ${}_{c}$	$H_{c}$	$H_{c}$/$\rho_{c}$
**R1**	0.133	0.320	2.186	0.130	−0.002	−0.014
**R2**	0.113	0.307	2.122	0.115	0.000	0.002
R2*	0.026	0.212	2.705	0.051	0.007	0.143
**R3**	0.205	0.358	1.922	0.202	0.000	0.002
**R4**	0.209	0.360	1.905	0.183	−0.001	−0.005
**R5**	0.044	0.241	2.457	0.071	0.003	0.048

Force constant $k^{a}$ in mdyn/Å, R(HB) in Å, *ρ_c_* in e/Å^3^ and *H_c_* in Hartree/Å^3^, normalized energy density *H_c_*/*ρ_c_* in Hartree/electron. Reference complexes **R1**–**R5** are shown in Figure 3. Calculated at the ωB97X-D/6-31++G(d,p) level of theory. R2* represents C–H⋯N bond.

**Table 2:** Comparison of the **AT** and **GC** base pair HBs in gas phase and DNA ${}^{a}$.

Base Pair	X–H Bond		Y⋯H Bond		X⋯Y Distance	
	d	k ${}^{a}$	d	k ${}^{a}$	d${}_{\mathrm{calc}}$	d${}_{\exp}{\;}^{b}$
	(Å)	(mdyn/Å)	(Å)	(mdyn/Å)	(Å)	(Å)
**AT1${}_{DNA}$**						
N–H⋯O	1.020	6.747	2.108	0.128	3.122	3.050
N⋯H–N	1.050	4.496	1.764	0.383	2.812	2.776
C–H⋯O	1.084	5.772	2.504	0.156	3.334	3.468
**AT2${}_{DNA}$**						
N–H⋯O	1.015	7.014	2.144	0.102	3.143	2.981
N⋯H–N	1.065	3.338	1.638	0.420	2.700	2.761
C–H⋯O	1.085	5.701	2.385	0.141	3.234	3.475
**AT3${}_{gas}$**						
N–H⋯O	1.022	6.554	1.903	0.201	2.921	-
N⋯H–N	1.047	4.673	1.777	0.318	2.824	-
C–H⋯O	1.087	5.635	2.738	0.060	3.570	-
**GC1${}_{DNA}$**						
N–H⋯O	1.020	6.508	1.855	0.267	2.866	2.880
N–H⋯N	1.035	5.508	1.831	0.511	2.851	2.912
O⋯H–N	1.030	5.663	1.748	0.282	2.763	2.852
**GC2${}_{DNA}$**						
N–H⋯O	1.027	6.096	1.758	0.387	2.784	2.789
N–H⋯N	1.038	5.379	1.855	0.517	2.892	2.875
O⋯H–N	1.025	6.241	1.868	0.230	2.888	2.839
**GC3${}_{gas}$**						
N–H⋯O	1.022	6.418	1.866	0.264	2.888	-
N–H⋯N	1.033	5.695	1.891	0.486	2.924	-
O⋯H–N	1.034	5.496	1.750	0.283	2.785	-

${}^{a}$ QM/MM calculations in DNA: the base pair **AT1${}_{DNA}$**, **AT2${}_{DNA}$**, **GC1${}_{DNA}$**, **GC2${}_{DNA}$**, the ωB97X-D/6-31++G(d,p)/AMBER level of theory; QM calculations in the gas phase: the base pair **AT3${}_{gas}$** and **GC3${}_{gas}$**, the ωB97X-D/6-31++G(d,p) level of theory. The left atomic symbol of the base pair label corresponds to the purine basis (A and G), and the right atomic symbol corresponds to the pyrimidine basis (T and C); the X and Y symbols correspond to the hydrogen donor and acceptor atoms, respectively. ${}^{b}$ Taken from the experimental X-ray structure [135].

**Table 3:** Comparison of NBO charges of atoms X–H⋯Y and energy densities of the X–H and the Y⋯H HBs of the **AT** and **GC** base pairs in gas phase and DNA ${}^{a}$.

	NBO Atomic Charge (e)			H${}_{c}$ (Hartree/Å${}^{3}$)	
Base Pair	q${}_{X}$	q${}_{H}$	q${}_{Y}$	X–H	Y⋯H
**AT1${}_{DNA}$**					
N–H⋯O	−0.836	0.449	−0.665	−3.3479	−0.0047
N⋯H–N	−0.605	0.477	−0.687	−3.0368	−0.0189
C–H⋯O	0.275	0.243	−0.671	−2.1986	0.0054
**AT2${}_{DNA}$**					
N–H⋯O	−0.834	0.454	−0.708	−3.3701	−0.0027
N⋯H–N	−0.606	0.477	−0.685	−2.8613	−0.0661
C–H⋯O	0.289	0.239	−0.692	−2.1885	0.0040
**AT3${}_{gas}$**					
N–H⋯O	−0.829	0.458	−0.672	−3.2986	−0.0020
N⋯H–N	−0.615	0.477	−0.688	−3.0631	−0.0169
C–H⋯O	0.271	0.237	−0.668	−2.1493	0.0061
**GC1${}_{DNA}$**					
N–H⋯O	−0.835	0.456	−0.702	−3.3229	0.0007
N–H⋯N	−0.658	0.472	−0.655	−3.2082	−0.0074
O⋯H–N	−0.754	0.471	−0.791	−3.2149	0.0027
**GC2${}_{DNA}$**					
N–H⋯O	−0.851	0.454	−0.696	−3.2736	0.0027
N–H⋯N	−0.668	0.472	−0.671	−3.1751	−0.0067
O⋯H–N	−0.722	0.466	−0.800	−3.2716	0.0000
**GC3${}_{gas}$**					
N–H⋯O	−0.860	0.458	−0.711	−3.2831	−0.0007
N–H⋯N	−0.677	0.467	−0.661	−3.2291	−0.0007
O⋯H–N	−0.686	0.468	−0.810	−3.1677	−0.0047

${}^{a}$ QM/MM calculations in DNA: the base pair **AT1${}_{DNA}$**, **AT2${}_{DNA}$**, **GC1${}_{DNA}$**, **GC2${}_{DNA}$**, the ωB97X-D/6-31++G(d,p)/AMBER level of theory. X and Y correspond to the hydrogen donor and acceptor atoms, respectively. The left atomic symbol of the base pair label and the *q_X_* charge in e correspond to the purine basis (A and G); the right atomic symbol of the base pair label and the *q_Y_* charge in e correspond to the pyrimidine basis (T and C).

## Data Availability

The data presented in this study are available in supplementary material.

## Supplementary Materials

The following are available online. Cartesian coordinates of all **NBP${}_{s}$** and **UBP${}_{s}$** investigated in this work; Table S1: Bond distances *R*, local mode force constants *k*${}^{a}$, local mode frequencies $\omega_{a}$ and bond strength orders BSO for all **UBP** and **NBP** BHs investigated in this work; Table S2: NBO charges of atoms X–H⋯Y and energy density parameters for all **UBP** and **NBP** BHs investigated in this work; specific note Note about the QM/MM calculations.

## Abbreviations

The following abbreviations are used in this manuscript:

HB	Hydrogen bond
NBP	natural base pair
UBP	Unnatural base pair
