# Supplementary material for: Hydrogen Bonding in Natural and Unnatural Base Pairs—A Local Vibrational Mode Study
Source: Molecules. 2021 Apr 14;26(8):2268. doi: 10.3390/molecules26082268 (PMC8071019; doi:10.3390/molecules26082268)
Supplement: Supplementary file 1 [file molecules-26-02268-s001.pdf]

Supplementary Materials: Hydrogen Bonding in Natural and Unnatural Base Pairs - A Local Vibrational Mode Study

Nassim Beiranvand, Marek Freindorf, and Elfi Kraka*

Computational and Theoretical Chemistry Group (CATCO),

Department of Chemistry, Southern Methodist University,

3215 Daniel Ave, Dallas, Texas 75275-0314, USA

E-mail: ekraka@smu.edu

Coordinates of base pairs calculated in this study:

AA1

N	-1.83065	-1.08014	0.06400
C	-2.81989	-1.98790	0.07725
N	-4.13335	-1.79201	0.05004
C	-4.42555	-0.48539	0.00349
C	-3.52166	0.56912	-0.01523
C	-2.15225	0.22664	0.01821
N	-4.17083	1.78843	-0.06498
C	-5.43780	1.47064	-0.07578
N	-5.65977	0.11371	-0.03604
N	-1.18558	1.15756	0.00702
H	-2.49574	-3.02490	0.11532
H	-6.25583	2.17632	-0.11201
H	-6.54834	-0.36112	-0.03577
H	-1.45544	2.12605	-0.04399
H	-0.19861	0.89779	0.02139
N	5.20640	1.06000	-0.00617
C	5.63651	-0.20951	-0.05740
N	4.91625	-1.32755	-0.07622
C	3.60639	-1.06503	-0.03852
C	3.00911	0.19054	0.01162
C	3.88770	1.28956	0.02819
N	1.63001	0.07938	0.04173
C	1.40450	-1.21134	0.01071
N	2.55664	-1.95124	-0.03833
N	3.45156	2.57030	0.05940
H	6.71527	-0.33386	-0.08489
H	0.40936	-1.64438	0.02185
H	2.63078	-2.95560	-0.06953
H	2.47921	2.76544	0.22533
H	4.13237	3.30140	0.18397

AA2

N	4.90766	-1.31067	-0.43343
C	5.67951	-0.20317	-0.16876
H	6.75815	-0.23860	-0.23045
N	4.98076	0.85229	0.15308
C	3.67129	0.41309	0.09432
C	2.44257	1.05232	0.33596
N	2.36035	2.36056	0.67800
H	3.20731	2.84866	0.92140

N	1.31707	0.33987	0.20035
C	1.39782	-0.95833	-0.15037
H	0.43990	-1.46599	-0.22158
N	2.49301	-1.66714	-0.40124
C	3.60031	-0.92914	-0.26607
H	5.22616	-2.22939	-0.69776
H	1.48722	2.70099	1.04736
H	-1.27731	2.64453	-0.49641
N	-1.33814	1.67820	-0.21936
H	-0.48668	1.13670	-0.05822
C	-2.56276	1.12532	-0.19908
C	-2.78767	-0.24469	0.06207
N	-3.62987	1.90889	-0.43589
C	-4.85331	1.36496	-0.40239
H	-5.67178	2.05184	-0.60087
N	-5.19674	0.10255	-0.15878
C	-4.11714	-0.65558	0.06406
N	-1.93836	-1.30798	0.31546
C	-2.73985	-2.32762	0.46947
H	-2.42412	-3.33936	0.68269
N	-4.06754	-2.00145	0.33248
H	-4.86084	-2.61819	0.40387

AA3

N	-3.05044	-2.10051	-0.00079
C	-4.42730	-2.08271	-0.00062
H	-5.00303	-2.99757	-0.00090
N	-4.92948	-0.87807	-0.00013
C	-3.81907	-0.05394	0.00003
C	-3.67407	1.34685	0.00047
N	-4.73530	2.17811	0.00102
H	-5.67087	1.80841	0.00050
N	-2.43957	1.86864	0.00047
C	-1.38771	1.03839	0.00007
H	-0.40236	1.49669	0.00012
N	-1.39593	-0.29704	-0.00035
C	-2.64035	-0.79049	-0.00036
H	-2.45536	-2.91312	-0.00117
H	-4.57758	3.17186	0.00081
N	4.10231	1.94958	-0.00063
C	2.78112	2.32557	-0.00076
H	2.48742	3.36600	-0.00110
N	1.95616	1.31308	-0.00045
C	2.78149	0.20204	-0.00008
C	2.52641	-1.18734	0.00038

N	1.29024	-1.71309	0.00054
H	0.44001	-1.14887	0.00023
N	3.57957	-2.02451	0.00069
C	4.81635	-1.51115	0.00055
H	5.62156	-2.24103	0.00084
N	5.18687	-0.23303	0.00013
C	4.12185	0.57654	-0.00017
H	4.91072	2.55066	-0.00082
H	1.22626	-2.71779	0.00089

AA4

N	5.09245	-1.40572	-0.17801
C	5.91880	-0.30686	-0.13873
H	6.99385	-0.40621	-0.19279
N	5.27430	0.82426	-0.03251
C	3.94508	0.44585	-0.00076
C	2.75108	1.18110	0.10669
N	2.74364	2.53102	0.18226
H	3.61561	3.02353	0.28480
N	1.59066	0.51231	0.11773
C	1.60603	-0.83315	0.02684
H	0.62321	-1.29921	0.04676
N	2.66355	-1.62990	-0.07923
C	3.80644	-0.93543	-0.08833
H	5.36374	-2.37311	-0.25527
H	1.88192	3.00458	0.39476
N	-5.68873	-0.18722	-0.11060
C	-5.59103	1.18090	-0.21454
H	-6.46858	1.80405	-0.31548
N	-4.36014	1.61653	-0.17783
C	-3.60331	0.46702	-0.04186
C	-2.21123	0.25311	0.05492
N	-1.32662	1.26342	0.03118
H	-1.67180	2.19935	-0.10281
N	-1.77365	-1.01255	0.17837
C	-2.67312	-2.00685	0.20625
H	-2.25571	-3.00522	0.30780
N	-3.99893	-1.93521	0.12585
C	-4.40659	-0.66587	0.00180
H	-6.52885	-0.74305	-0.11529
H	-0.32206	1.07780	0.07341

AC1

N	-4.60569	-0.82759	0.00002

C	-4.98909	0.49356	0.00021
H	-6.03141	0.77997	0.00030
N	-3.98108	1.32428	-0.00006
C	-2.87060	0.50118	-0.00000
C	-1.48594	0.77846	-0.00003
N	-0.98567	2.01948	-0.00007
H	-1.62469	2.79718	-0.00007
H	0.03066	2.17207	-0.00012
N	-0.64632	-0.27568	-0.00005
C	-1.14111	-1.52638	-0.00005
H	-0.38950	-2.31191	-0.00007
N	-2.41387	-1.90116	-0.00003
C	-3.23366	-0.84013	-0.00000
H	-5.20313	-1.63883	0.00000
H	1.13322	-0.10380	-0.00009
N	1.97653	2.24303	-0.00003
H	2.51500	3.10467	-0.00001
C	2.75644	1.21749	-0.00001
N	2.17719	-0.04374	-0.00006
C	2.83195	-1.24508	-0.00007
O	2.27025	-2.32767	-0.00001
N	4.22005	-1.14687	0.00006
H	4.71568	-2.02465	0.00011
C	4.88215	0.05474	0.00010
H	5.96430	-0.00285	0.00018
C	4.21556	1.22302	0.00006
H	4.74578	2.16601	0.00010

AC2

N	4.61301	-0.76012	-0.00020
C	4.96941	0.56672	0.00021
H	6.00386	0.87887	0.00025
N	3.93561	1.36873	0.00025
C	2.84928	0.52414	0.00004
C	1.44252	0.82765	-0.00005
N	0.83853	1.96949	-0.00005
H	1.52680	2.71813	-0.00002
H	-0.98752	2.19510	-0.00011
N	0.68986	-0.34627	-0.00005
C	1.19429	-1.60369	0.00001
H	0.44130	-2.38691	-0.00006
N	2.46289	-1.91429	0.00003
C	3.24494	-0.80073	0.00008
H	5.22486	-1.56078	-0.00045
H	-0.34481	-0.23821	-0.00009

N	-2.02846	2.24994	-0.00010
H	-2.48042	3.14850	-0.00010
C	-2.76294	1.13572	-0.00008
N	-2.11908	-0.02928	-0.00008
C	-2.78532	-1.21569	-0.00010
O	-2.23673	-2.31112	0.00011
N	-4.19099	-1.15140	0.00005
H	-4.67016	-2.03962	0.00014
C	-4.87085	0.02416	0.00004
H	-5.95287	-0.04128	0.00012
C	-4.20540	1.20189	-0.00005
H	-4.73242	2.14663	-0.00004

AC3

N	2.34278	2.52579	0.32962
H	1.55819	3.04925	0.68190
H	3.26772	2.89255	0.48852
C	2.20135	1.18259	0.19415
C	3.30443	0.33052	0.01715
N	4.66700	0.56129	-0.02993
C	5.17880	-0.62758	-0.20621
H	6.23486	-0.84195	-0.29207
N	4.23500	-1.62535	-0.27671
H	4.39461	-2.61236	-0.40278
C	3.01122	-1.02156	-0.13123
N	1.80062	-1.58682	-0.12015
C	0.83715	-0.68944	0.05388
H	-0.18970	-1.04751	0.08384
N	0.97560	0.64587	0.20506
H	-0.79926	1.48925	0.05255
N	-1.75932	1.82641	-0.07641
H	-1.91809	2.76872	-0.38835
H	-4.33315	2.43020	-0.57780
C	-4.11243	1.39318	-0.36198
C	-2.77188	0.93634	-0.08075
N	-2.47885	-0.32353	0.18869
C	-3.45897	-1.27424	0.21391
O	-3.28131	-2.45947	0.44584
N	-4.77828	-0.83496	-0.05465
H	-5.48540	-1.55460	-0.03135
C	-5.08653	0.45428	-0.33433
H	-6.13002	0.67681	-0.52731

AG1

N	3.84769	-1.97215	-0.23165

C	5.19289	-1.67787	-0.20267
H	5.94152	-2.45091	-0.30419
N	5.44119	-0.40658	-0.04399
C	4.18769	0.17113	0.03335
C	3.76485	1.50477	0.19682
N	4.63646	2.52382	0.32123
H	5.62743	2.35088	0.30117
N	2.44958	1.76588	0.22730
C	1.58897	0.74799	0.10535
H	0.53157	0.99787	0.12718
N	1.86377	-0.54972	-0.04839
C	3.18243	-0.78178	-0.08119
H	3.42880	-2.87921	-0.36006
H	4.28331	3.45898	0.43685
N	-5.09348	1.30166	-0.00784
C	-4.16522	0.28181	0.02272
C	-4.38995	2.37613	-0.22724
H	-4.78412	3.37846	-0.31456
N	-3.03917	2.11684	-0.34400
H	-2.29933	2.77867	-0.51664
C	-2.88336	0.76995	-0.18565
N	-1.70351	0.10126	-0.22047
C	-1.84782	-1.19088	-0.04833
N	-3.06841	-1.78307	0.15810
C	-4.33503	-1.13259	0.21804
O	-5.33596	-1.79823	0.40654
H	-3.13141	-2.78987	0.23174
N	-0.76528	-2.01351	-0.10905
H	-0.81763	-2.90201	0.36280
H	0.15299	-1.55996	-0.11257

AG2

N	-4.94129	1.72597	-0.45423
C	-5.94071	0.80532	-0.23953
H	-6.98426	1.07513	-0.32100
N	-5.49460	-0.38455	0.06231
C	-4.12078	-0.23761	0.04325
C	-3.06765	-1.13669	0.28801
N	-3.28464	-2.43696	0.59096
H	-4.22496	-2.73907	0.78886
N	-1.81037	-0.68239	0.19640
C	-1.60093	0.61175	-0.11663
H	-0.55415	0.90147	-0.15831
N	-2.50769	1.54849	-0.36573
C	-3.75256	1.06621	-0.27352

H	-5.04758	2.69839	-0.69659
H	-2.51589	-2.98608	0.93884
N	5.05539	1.45839	0.01653
C	4.16483	0.40703	-0.04695
C	4.32536	2.48407	0.35211
H	4.68663	3.49214	0.49682
N	2.99291	2.16242	0.51439
H	2.23925	2.77877	0.77362
C	2.87845	0.82583	0.26075
N	1.72702	0.10941	0.30014
C	1.91012	-1.15945	0.02309
N	3.13934	-1.68482	-0.28658
C	4.37619	-0.98085	-0.35879
O	5.39013	-1.58868	-0.64666
H	3.23447	-2.68035	-0.43816
N	0.86196	-2.02745	0.07320
H	0.91342	-2.86077	-0.49139
H	-0.06803	-1.60361	0.15826

AT1

N	-2.87759	2.10263	-0.00002
C	-1.51063	2.03980	-0.00001
N	-1.06691	0.80856	0.00003
C	-2.20009	0.01722	-0.00005
C	-2.39893	-1.38155	-0.00009
N	-1.39188	-2.26751	-0.00003
N	-3.66632	-1.83257	-0.00015
C	-4.67073	-0.94703	-0.00019
N	-4.61083	0.38355	-0.00016
C	-3.34278	0.80688	-0.00009
H	-3.44803	2.93320	-0.00002
H	-0.87432	2.91472	0.00001
H	-0.41038	-1.99558	0.00002
H	-1.63269	-3.24523	-0.00007
H	-5.66818	-1.37812	-0.00024
N	3.76617	1.48161	0.00006
C	4.39695	0.25805	0.00005
C	3.71718	-0.90649	0.00008
C	4.36585	-2.25687	0.00005
C	2.25444	-0.83569	0.00013
O	1.52487	-1.82580	0.00008
N	1.70899	0.43467	0.00012
C	2.39084	1.62664	0.00014
O	1.84649	2.71991	0.00003
H	4.29439	2.34062	0.00001

H	5.48087	0.29639	-0.00000
H	5.45533	-2.16864	0.00001
H	4.06044	-2.83216	0.87884
H	4.06037	-2.83216	-0.87871
H	0.66726	0.51194	0.00010

AT2

N	-2.49525	-2.31602	-0.00018
C	-1.16649	-1.98913	-0.00012
N	-0.97067	-0.69512	-0.00012
C	-2.23564	-0.13885	-0.00013
C	-2.70185	1.19437	-0.00012
N	-1.88517	2.25934	-0.00007
N	-4.03216	1.39167	-0.00014
C	-4.84599	0.32808	-0.00020
N	-4.52932	-0.96555	-0.00019
C	-3.20336	-1.13543	-0.00017
H	-2.89327	-3.24170	-0.00022
H	-0.37069	-2.72233	-0.00010
H	-0.87077	2.18199	0.00002
H	-2.31015	3.17213	-0.00001
H	-5.90806	0.55773	-0.00017
N	3.29724	1.82607	0.00025
C	4.29064	0.86940	0.00026
C	4.02171	-0.45088	0.00018
C	5.07091	-1.51980	0.00016
C	2.61215	-0.86133	0.00011
O	2.23378	-2.02551	-0.00002
N	1.68672	0.17694	0.00008
C	1.95425	1.51613	0.00016
O	1.08730	2.38511	0.00016
H	3.51937	2.80965	0.00032
H	5.30366	1.25660	0.00032
H	6.07434	-1.08635	0.00022
H	4.96548	-2.16301	0.87862
H	4.96555	-2.16293	-0.87836
H	0.67826	-0.09533	0.00002

AT3

N	4.85027	-1.05211	-0.00001
C	5.32167	0.24025	-0.00004
H	6.38090	0.45565	-0.00011
N	4.37234	1.13732	0.00008
C	3.20939	0.39110	0.00012

C	1.84761	0.76014	0.00021
N	1.43477	2.03477	0.00034
H	2.12202	2.76997	0.00013
H	0.43958	2.25736	0.00014
N	0.93845	-0.23332	0.00020
C	1.34677	-1.51565	0.00012
H	0.54354	-2.24834	0.00011
N	2.59080	-1.97476	0.00004
C	3.48081	-0.97218	0.00004
H	5.39191	-1.90174	-0.00005
N	-3.61960	1.67774	-0.00015
C	-4.51494	0.62799	-0.00018
H	-5.56110	0.91377	-0.00023
C	-4.11580	-0.65801	-0.00013
C	-5.05223	-1.82681	-0.00017
H	-6.09425	-1.49649	-0.00022
H	-4.88180	-2.45624	-0.87838
H	-4.88188	-2.45622	0.87808
C	-2.67140	-0.92852	-0.00005
O	-2.18377	-2.04885	-0.00005
N	-1.85044	0.19703	-0.00004
H	-0.81594	0.03157	0.00001
C	-2.25333	1.50174	-0.00010
O	-1.48047	2.45745	-0.00012
H	-3.93646	2.63489	-0.00021

AT4

N	4.70344	-1.27836	0.00061
C	5.41821	-0.10326	0.00036
H	6.49911	-0.09914	0.00055
N	4.66381	0.96322	0.00020
C	3.37798	0.45784	-0.00010
C	2.11603	1.07786	-0.00053
N	1.96601	2.41740	-0.00105
H	2.77861	3.01076	-0.00014
H	1.04554	2.82508	-0.00043
N	1.03090	0.28912	-0.00053
C	1.17372	-1.05416	-0.00017
H	0.23771	-1.60634	-0.00017
N	2.30786	-1.73908	0.00021
C	3.37639	-0.93312	0.00024
H	5.06862	-2.21775	0.00091
N	-4.41154	-1.21411	0.00003
C	-4.71093	0.11304	0.00030
H	-5.76513	0.37001	0.00048

C	-3.72963	1.04793	0.00033
C	-3.97663	2.52729	0.00061
H	-5.04929	2.74020	0.00074
H	-3.53170	3.00039	0.88117
H	-3.53185	3.00070	-0.87985
C	-2.39166	0.50668	0.00008
O	-1.40752	1.38626	0.00007
N	-2.09805	-0.77032	-0.00020
H	-0.51440	0.90551	-0.00014
C	-3.09612	-1.71178	-0.00025
O	-2.91495	-2.91881	-0.00048
H	-5.13874	-1.91403	-0.00001

ATWC

N	4.95968	0.54808	0.00013
C	5.21162	-0.80443	0.00030
H	6.22082	-1.19156	0.00046
N	4.12727	-1.53252	-0.00039
C	3.10336	-0.60473	-0.00010
C	1.69913	-0.74457	-0.00014
N	1.08114	-1.93255	-0.00042
H	1.63738	-2.77129	-0.00028
H	0.06154	-1.98610	-0.00027
N	0.96598	0.38568	-0.00004
C	1.58069	1.58272	0.00009
H	0.91032	2.43853	0.00014
N	2.88328	1.83077	0.00011
C	3.59584	0.69495	0.00002
H	5.63397	1.29678	0.00026
N	-3.83611	1.58763	-0.00013
C	-4.55451	0.41488	-0.00014
H	-5.63284	0.53180	-0.00037
C	-3.96085	-0.79586	0.00010
C	-4.70580	-2.09569	0.00008
H	-5.78600	-1.92812	-0.00003
H	-4.44322	-2.69191	-0.87862
H	-4.44339	-2.69184	0.87889
C	-2.49721	-0.83131	0.00021
O	-1.84644	-1.87670	0.00021
N	-1.85711	0.39380	0.00033
H	-0.80919	0.38959	0.00017
C	-2.45224	1.63351	0.00022
O	-1.83349	2.68389	-0.00033
H	-4.29896	2.48357	-0.00049

AU1

N	-2.36878	2.22436	0.00005
C	-1.01738	2.01117	-0.00012
N	-0.71187	0.73846	-0.00002
C	-1.92558	0.07669	-0.00004
C	-2.27824	-1.29164	-0.00009
N	-1.37627	-2.28409	-0.00009
N	-3.58792	-1.59923	-0.00005
C	-4.48806	-0.60805	0.00001
N	-4.28113	0.70775	0.00004
C	-2.97406	0.98783	0.00001
H	-2.84416	3.11278	0.00011
H	-0.28913	2.81111	-0.00020
H	-0.37068	-2.12356	-0.00008
H	-1.72432	-3.22899	-0.00010
H	-5.52713	-0.92604	0.00001
N	4.17221	0.87643	0.00017
C	4.66471	-0.40407	0.00010
C	3.84767	-1.47494	-0.00001
C	2.40599	-1.26775	-0.00009
O	1.57210	-2.16919	-0.00020
N	2.00470	0.05951	0.00001
C	2.81459	1.16728	0.00018
O	2.39855	2.31420	0.00014
H	4.78683	1.67617	0.00023
H	5.74505	-0.48960	0.00013
H	0.97757	0.25338	-0.00006
H	4.22639	-2.48683	-0.00006

AU2

N	2.37129	2.22042	-0.00013
C	1.01945	2.00959	-0.00048
N	0.71242	0.73721	-0.00046
C	1.92465	0.07370	-0.00013
C	2.27435	-1.29449	0.00003
N	1.36864	-2.28529	-0.00013
N	3.58237	-1.60614	0.00034
C	4.48516	-0.61683	0.00044
N	4.28103	0.69922	0.00038
C	2.97480	0.98303	0.00006
H	2.84756	3.10839	-0.00005
H	0.29015	2.80898	-0.00072
H	0.36544	-2.11963	-0.00033
H	1.71278	-3.23149	0.00004
H	5.52342	-0.93727	0.00084
N	-3.76580	-1.39973	0.00037

C	-4.66931	-0.36394	0.00068
C	-4.26918	0.92104	0.00045
C	-2.84152	1.22724	-0.00022
O	-2.36106	2.35111	-0.00028
N	-2.01057	0.10641	-0.00040
C	-2.39608	-1.20277	-0.00015
O	-1.61311	-2.14634	-0.00040
H	-4.06792	-2.36197	0.00055
H	-5.71289	-0.65589	0.00112
H	-0.98092	0.28635	-0.00079
H	-4.97378	1.74035	0.00074

AU3

N	4.60728	0.80228	0.00072
C	4.97241	-0.52399	0.00082
H	6.01069	-0.82462	0.00097
N	3.95343	-1.34117	0.00049
C	2.85488	-0.50301	0.00045
C	1.46787	-0.76025	0.00026
N	0.95348	-1.99773	0.00008
H	1.57895	-2.78614	0.00012
H	-0.05586	-2.13910	-0.00005
N	0.64201	0.30333	0.00023
C	1.15291	1.54858	0.00038
H	0.41189	2.34414	0.00034
N	2.43005	1.90501	0.00055
C	3.23597	0.83380	0.00059
H	5.21607	1.60518	0.00081
N	-4.06276	-1.22595	-0.00078
C	-4.86702	-0.11064	-0.00105
H	-5.93316	-0.30451	-0.00135
C	-4.34779	1.13018	-0.00093
C	-2.89674	1.30324	-0.00053
O	-2.31723	2.37686	-0.00036
N	-2.17141	0.10845	-0.00027
H	-1.12593	0.18537	0.00005
C	-2.68132	-1.15706	-0.00037
O	-1.99412	-2.17450	-0.00013
H	-4.45181	-2.15631	-0.00086
H	-4.97163	2.01257	-0.00113

AU4

N	-3.27468	2.16504	0.00010
C	-1.90608	2.25647	-0.00002

N	-1.31034	1.09327	-0.00012
C	-2.35013	0.17720	-0.00012
C	-2.39497	-1.23704	-0.00018
N	-1.30493	-2.01667	-0.00022
N	-3.60432	-1.82836	-0.00030
C	-4.70299	-1.06536	-0.00028
N	-4.79406	0.26293	-0.00024
C	-3.58134	0.82593	-0.00017
H	-3.94060	2.92102	0.00025
H	-1.40317	3.21352	0.00007
H	-0.35263	-1.66260	-0.00015
H	-1.44900	-3.01339	-0.00031
H	-5.64476	-1.60768	-0.00046
N	4.31380	1.23156	0.00036
C	2.98068	1.56937	0.00035
C	2.00640	0.64055	0.00022
C	2.37601	-0.76490	0.00005
O	1.59606	-1.70898	-0.00008
N	3.75075	-1.00855	0.00009
C	4.77036	-0.07517	0.00023
O	5.94998	-0.36519	0.00024
H	5.03433	1.93749	0.00047
H	2.77325	2.63298	0.00049
H	4.04300	-1.97790	-0.00002
H	0.95000	0.89325	0.00027

AU5

N	2.18642	-1.96548	-0.02452
C	1.01747	-1.25257	0.07242
N	1.20929	0.04326	0.11132
C	2.58393	0.18478	0.03565
C	3.43353	1.30557	0.03676
N	2.96042	2.57416	0.09469
N	4.75607	1.11407	-0.04269
C	5.21777	-0.14326	-0.12395
N	4.52752	-1.27997	-0.13348
C	3.21237	-1.05418	-0.04938
H	2.28118	-2.96760	-0.06958
H	0.04540	-1.73383	0.10901
H	1.99204	2.72794	0.31932
H	3.62372	3.31866	0.23446
H	6.29804	-0.23811	-0.18800
N	-4.34446	1.42181	-0.09538
C	-2.97632	1.54821	-0.05404
C	-2.15961	0.48124	0.02828

C	-2.74134	-0.85328	0.07207
O	-2.12207	-1.90537	0.14142
N	-4.13939	-0.87536	0.02698
C	-4.99951	0.20262	-0.05601
O	-6.20950	0.09995	-0.09155
H	-4.94474	2.23010	-0.15686
H	-2.60363	2.56496	-0.09292
H	-4.58007	-1.78631	0.05607
H	-1.08021	0.58534	0.06042

AU6

N	4.35874	-1.45096	0.00052
C	5.16141	-0.33410	0.00056
H	6.23944	-0.41281	0.00085
N	4.49092	0.78702	0.00045
C	3.17024	0.38167	-0.00003
C	1.95961	1.09693	-0.00049
N	1.91296	2.44379	-0.00078
H	2.76888	2.97279	0.00011
H	1.02672	2.92148	-0.00039
N	0.81707	0.39350	-0.00069
C	0.85673	-0.95666	-0.00051
H	-0.11874	-1.43563	-0.00068
N	1.93477	-1.72675	-0.00012
C	3.06205	-1.00507	0.00012
H	4.65087	-2.41556	0.00070
N	-4.74220	-0.66707	0.00025
C	-4.93282	0.67610	0.00058
H	-5.96022	1.02191	0.00087
C	-3.86675	1.51107	0.00054
C	-2.57648	0.88104	0.00013
O	-1.52684	1.67755	0.00009
N	-2.39058	-0.41765	-0.00019
H	-0.67398	1.12773	-0.00020
C	-3.46509	-1.27007	-0.00015
O	-3.38966	-2.48706	-0.00043
H	-5.52111	-1.30920	0.00027
H	-3.96500	2.58679	0.00079

AUWC

N	-4.60684	-0.81094	-0.00012
C	-4.97569	0.51439	-0.00017
H	-6.01477	0.81232	-0.00024
N	-3.95881	1.33411	-0.00015

C	-2.85781	0.49904	-0.00005
C	-1.47097	0.76115	0.00003
N	-0.96031	1.99886	-0.00002
H	-1.58824	2.78542	0.00000
H	0.05020	2.14371	0.00015
N	-0.64212	-0.30102	0.00012
C	-1.15051	-1.54708	0.00013
H	-0.40835	-2.34146	0.00020
N	-2.42623	-1.90769	0.00005
C	-3.23553	-0.83841	-0.00004
H	-5.21349	-1.61542	-0.00014
N	4.25157	-1.08117	-0.00008
C	4.86549	0.14446	-0.00019
H	5.94912	0.12577	-0.00030
C	4.15509	1.28886	-0.00015
C	2.70037	1.22106	0.00004
O	1.96166	2.20384	0.00011
N	2.16960	-0.05944	0.00014
H	1.12487	-0.14864	0.00028
C	2.87097	-1.24135	0.00003
O	2.34963	-2.34213	0.00027
H	4.78524	-1.93707	-0.00006
H	4.62850	2.26003	-0.00024

CC

H	0.16249	1.28633	0.00013
N	-0.75500	1.75887	0.00019
H	-0.78662	2.76396	0.00009
C	-1.87599	1.03497	0.00008
C	-3.16491	1.68746	-0.00002
H	-3.25638	2.76537	-0.00003
C	-4.25397	0.88618	-0.00012
N	-1.75729	-0.29095	0.00006
N	-4.11172	-0.46409	-0.00009
H	-5.26853	1.26815	-0.00021
H	-4.91077	-1.08021	-0.00023
C	-2.85317	-1.09926	-0.00001
O	-2.80904	-2.32286	-0.00003
H	-0.16249	-1.28638	0.00014
N	0.75501	-1.75890	0.00015
H	0.78663	-2.76398	0.00012
C	1.87599	-1.03499	0.00006
N	1.75728	0.29094	0.00001
C	2.85317	1.09926	-0.00004
O	2.80901	2.32287	-0.00002

N	4.11172	0.46411	-0.00013
H	4.91075	1.08025	-0.00009
C	4.25398	-0.88616	-0.00009
H	5.26854	-1.26812	-0.00017
C	3.16493	-1.68745	0.00000
H	3.25643	-2.76536	0.00002

CU

N	-3.51417	-1.09432	-0.35994
C	-4.16733	0.04526	0.02138
H	-5.24977	-0.01120	0.02506
C	-3.49254	1.16105	0.35980
H	-3.99347	2.07208	0.65322
C	-2.03857	1.14908	0.32078
O	-1.34088	2.13343	0.56821
N	-1.45930	-0.06101	-0.02707
H	-0.42374	-0.12453	0.04715
C	-2.12540	-1.20403	-0.42266
O	-1.58430	-2.22048	-0.80185
N	3.48278	-1.14493	0.16916
C	4.13592	-0.06902	-0.33143
H	5.19168	-0.19438	-0.54369
C	3.47490	1.09365	-0.53828
H	3.97674	1.96802	-0.93042
C	2.07004	1.10400	-0.21340
N	1.35167	2.22431	-0.38984
H	0.38954	2.25968	-0.03744
H	1.80199	3.07341	-0.68413
N	1.43504	0.03592	0.25167
C	2.10926	-1.12918	0.50375
O	1.61227	-2.12885	0.99011
H	3.95840	-2.01717	0.34689
H	-4.01785	-1.92337	-0.63594

GC1

N	3.08050	2.33454	-0.01114
C	1.72479	2.56548	-0.01854
H	1.31596	3.56544	-0.02668
N	1.02866	1.46129	-0.01472
C	1.97005	0.45365	-0.00393
C	1.81141	-0.97325	-0.00596
O	0.80395	-1.65809	-0.01879
N	3.08479	-1.60313	0.00053
H	3.03930	-2.61221	-0.06179

C	4.30123	-0.97132	0.01132
N	5.40970	-1.76845	-0.03122
H	5.36196	-2.70108	0.34738
H	6.28013	-1.28110	0.11810
N	4.44051	0.32896	0.01672
C	3.25209	0.98023	-0.00237
H	3.81829	3.02146	-0.01354
N	-4.67684	-1.39944	-0.01211
C	-3.35590	-1.71618	-0.02436
H	-3.10606	-2.77117	-0.04154
C	-2.42228	-0.73857	-0.01496
H	-1.36142	-0.95854	-0.02337
C	-2.92722	0.61229	0.00769
N	-2.04287	1.63019	0.01736
H	-1.03830	1.47943	0.00421
H	-2.41879	2.56435	0.03086
N	-4.21274	0.92092	0.02023
C	-5.15398	-0.06837	0.01137
O	-6.36428	0.10895	0.02182
H	-5.39147	-2.11129	-0.01892

GCWC

N	4.65454	0.47938	-0.00012
C	4.95310	-0.87010	-0.00017
H	5.97463	-1.22296	-0.00026
N	3.89217	-1.62433	-0.00001
C	2.83638	-0.73249	-0.00000
C	1.42433	-0.94747	0.00004
O	0.80561	-2.01759	0.00008
N	0.72133	0.26296	0.00011
H	-0.30845	0.17656	0.00014
C	1.28105	1.51547	0.00011
N	0.42683	2.55624	0.00013
H	-0.58818	2.44275	0.00009
H	0.83169	3.47646	0.00007
N	2.58380	1.72954	0.00003
C	3.29229	0.58208	-0.00001
N	-4.31995	0.91212	-0.00011
C	-4.92611	-0.30405	-0.00013
H	-6.00979	-0.30615	-0.00020
C	-4.18503	-1.43432	-0.00005
H	-4.64824	-2.41183	-0.00005
C	-2.75034	-1.27590	0.00002
N	-1.95312	-2.34270	0.00010
H	-0.92362	-2.22911	0.00018

H	-2.34596	-3.26868	0.00014
N	-2.17710	-0.07035	0.00003
C	-2.92687	1.05881	0.00000
O	-2.45834	2.19863	-0.00005
H	5.29981	1.25279	-0.00017
H	-4.85234	1.76965	-0.00015

GG

N	-5.50344	0.78696	0.08274
C	-5.96515	-0.51417	0.10888
H	-7.02120	-0.73949	0.15431
N	-5.00404	-1.39262	0.07312
C	-3.84773	-0.63879	0.02009
C	-2.46731	-1.03231	-0.03285
O	-1.96444	-2.15152	-0.04304
N	-1.63365	0.10287	-0.07723
H	-0.62669	-0.11529	-0.10163
C	-2.03602	1.40699	-0.06774
N	-1.06121	2.34557	-0.13708
H	-0.07195	2.12987	-0.06879
H	-1.35118	3.30404	-0.04573
N	-3.29903	1.78009	-0.01428
C	-4.13940	0.72073	0.02533
H	-6.04769	1.63417	0.10010
O	2.03519	2.18329	0.00287
C	2.72565	1.17213	0.00039
N	4.13535	1.27058	0.04798
H	4.48457	2.21603	0.13937
N	6.34094	0.52305	0.15470
H	6.66828	1.39621	-0.22801
H	6.95889	-0.26369	0.02365
C	5.01261	0.21225	0.05872
N	4.64274	-1.04138	0.02839
C	3.29534	-1.18928	-0.00409
C	2.33010	-0.19889	-0.03427
N	1.07272	-0.74774	-0.07328
C	1.25747	-2.03997	-0.06723
H	0.45470	-2.76638	-0.08708
N	2.59529	-2.36340	-0.02726
H	2.99314	-3.28962	-0.01286

GU1

N	-5.03325	0.04641	0.13065
C	-5.10655	-1.33216	0.11128

H	-6.05390	-1.84960	0.16253
N	-3.93719	-1.89993	0.02682
C	-3.04466	-0.84769	-0.01331
C	-1.61187	-0.83602	-0.09744
O	-0.81688	-1.76760	-0.14993
N	-1.13210	0.49017	-0.11371
H	-0.11642	0.60704	-0.15322
C	-1.89075	1.62479	-0.04427
N	-1.20896	2.80023	-0.10360
H	-0.20809	2.80553	0.02516
H	-1.72934	3.63306	0.11549
N	-3.20030	1.62630	0.04382
C	-3.70962	0.37107	0.05089
H	-5.79546	0.70254	0.18841
H	4.01565	1.84825	0.05641
N	3.83740	0.85183	0.03669
C	2.50946	0.44666	-0.02619
O	1.62039	1.29980	-0.05191
C	2.30343	-0.98742	-0.05598
H	1.28919	-1.37759	-0.10302
C	3.38029	-1.79682	-0.02135
H	3.29330	-2.87663	-0.04009
N	4.66167	-1.30637	0.04036
H	5.45864	-1.92486	0.06491
C	4.96172	0.04336	0.07254
O	6.09485	0.47494	0.12674

GU2

N	-1.77136	-2.21109	0.01331
C	-0.46111	-1.79990	0.00555
H	0.38327	-2.47675	0.00556
N	-0.35359	-0.49635	-0.00299
C	-1.65182	-0.02907	-0.00033
C	-2.16916	1.31329	-0.01968
O	-1.60091	2.38592	-0.04528
N	-3.59673	1.27718	-0.01767
H	-4.02319	2.19098	-0.09999
C	-4.38130	0.15618	0.00322
N	-5.73777	0.35164	-0.04842
H	-6.11694	1.18793	0.36817
H	-6.27415	-0.48660	0.11876
N	-3.90451	-1.05971	0.02443
C	-2.54668	-1.08656	0.00845
H	-2.11006	-3.16004	0.01902
H	1.50894	0.07358	0.00225

N	2.54338	0.08876	0.00315
C	3.19576	-1.14215	-0.01269
O	2.56288	-2.19167	-0.02225
C	4.65207	-1.06946	-0.01547
H	5.21068	-1.99421	-0.02865
C	5.25570	0.13347	-0.00164
H	6.33311	0.25207	-0.00296
N	4.53059	1.29470	0.01557
H	4.97988	2.19739	0.02687
C	3.13744	1.33439	0.01980
O	2.53215	2.38555	0.03703

GU3

N	2.24339	2.10805	-0.56453
C	3.53731	2.56480	-0.40267
H	3.79791	3.60006	-0.57011
N	4.36116	1.62502	-0.03735
C	3.57966	0.49086	0.04114
C	3.92729	-0.86186	0.38287
O	4.99125	-1.35495	0.70508
N	2.77158	-1.69234	0.30221
H	2.95362	-2.65522	0.55303
C	1.49861	-1.30317	-0.03047
N	0.53405	-2.25249	-0.00570
H	0.78722	-3.22588	0.01529
H	-0.38410	-2.02282	-0.38874
N	1.18993	-0.06036	-0.33110
C	2.26064	0.77124	-0.28374
H	1.44361	2.63410	-0.87737
H	-4.65640	-1.50425	-0.74039
N	-4.16404	-0.70594	-0.35882
C	-2.77123	-0.76060	-0.36947
O	-2.20846	-1.75120	-0.82343
C	-2.11105	0.40959	0.18438
H	-1.02633	0.43854	0.17464
C	-2.86475	1.41128	0.67659
H	-2.43494	2.30583	1.11181
N	-4.23764	1.36702	0.66065
H	-4.79149	2.12314	1.03416
C	-4.96242	0.30911	0.13722
O	-6.17559	0.28067	0.12023

HC

N	-4.19611	-1.12688	0.00107

C	-4.85853	0.05854	0.00330
H	-5.94129	0.00861	0.00522
C	-4.17617	1.22685	0.00311
H	-4.68877	2.17937	0.00497
C	-2.73633	1.13765	0.00050
N	-1.98388	2.24161	0.00059
H	-2.41155	3.15155	0.00178
H	-0.95645	2.16250	-0.00147
N	-2.10877	-0.03470	-0.00198
C	-2.79212	-1.21261	-0.00144
O	-2.25734	-2.31405	-0.00272
H	-4.68922	-2.00760	0.00149
H	0.48614	-2.43182	-0.00073
C	1.21932	-1.63027	-0.00058
N	2.49533	-1.90446	0.00130
N	0.69453	-0.37996	-0.00254
H	-0.34237	-0.28107	-0.00329
C	1.42088	0.81523	-0.00278
O	0.84006	1.90330	-0.00467
C	2.83216	0.54860	-0.00049
C	3.25032	-0.77735	0.00129
N	3.91138	1.40503	0.00042
C	4.95127	0.61607	0.00229
H	5.98200	0.94122	0.00337
N	4.61567	-0.71812	0.00290
H	5.23802	-1.51068	0.00426

HU1

N	4.94944	0.50309	-0.00029
C	5.16535	-0.85704	-0.00035
H	6.16513	-1.26845	-0.00052
N	4.06761	-1.56047	-0.00031
C	3.06264	-0.61192	-0.00007
C	1.65838	-0.69196	0.00011
O	1.04451	-1.85087	0.00010
N	0.96068	0.45105	0.00033
H	-0.82393	0.54370	0.00035
C	1.60696	1.63409	0.00035
N	2.91424	1.83525	0.00018
C	3.59226	0.68059	-0.00002
H	5.64102	1.23587	-0.00036
O	-1.64144	-1.77772	0.00043
H	0.05622	-1.75321	0.00030
C	-2.39124	-0.79608	0.00022
N	-1.86614	0.47778	0.00028

C	-2.57005	1.66078	0.00012
O	-2.04739	2.76035	-0.00015
N	-3.94342	1.48443	-0.00031
H	-4.48893	2.33286	-0.00059
C	-4.54950	0.25046	-0.00035
H	-5.63393	0.26701	-0.00065
C	-3.84748	-0.90196	-0.00006
C	-4.46778	-2.26579	-0.00010
H	-5.55867	-2.19899	-0.00034
H	-4.15103	-2.83477	-0.87883
H	-4.15140	-2.83465	0.87884
H	0.96591	2.51159	0.00051

HU2

N	-4.95115	0.33571	0.00003
C	-5.07852	-1.03444	-0.00010
H	-6.04747	-1.51318	-0.00014
N	-3.93094	-1.65590	-0.00015
C	-2.99596	-0.64479	-0.00006
C	-1.56437	-0.68724	-0.00006
O	-0.82573	-1.68139	-0.00016
N	-1.02555	0.59726	0.00005
H	0.01507	0.63431	0.00011
C	-1.73351	1.75428	0.00015
N	-3.03560	1.83019	0.00015
C	-3.61167	0.60277	0.00005
H	-5.68809	1.02328	0.00009
O	1.77236	-1.87283	-0.00005
H	0.77117	-1.73667	-0.00013
C	2.43115	-0.74348	-0.00007
N	1.77441	0.39857	-0.00023
C	2.42955	1.60151	-0.00064
O	1.85672	2.68323	0.00043
N	3.82407	1.53689	0.00002
H	4.30550	2.42424	0.00032
C	4.51381	0.36010	0.00017
H	5.59549	0.44005	0.00048
C	3.87133	-0.83314	0.00001
C	4.56354	-2.16349	0.00016
H	5.64905	-2.03460	0.00030
H	4.28345	-2.74997	0.87980
H	4.28369	-2.75003	-0.87951
H	-1.13344	2.65896	0.00023

TC

N	3.88408	0.83932	-0.26814
C	4.41124	-0.27598	0.29107
H	5.47780	-0.26704	0.48547
C	3.61865	-1.33576	0.57476
H	4.01817	-2.23962	1.01472
C	2.21744	-1.19443	0.26516
N	1.37166	-2.20634	0.51762
H	0.40552	-2.14363	0.18018
H	1.72026	-3.08421	0.86128
N	1.70790	-0.08726	-0.25855
C	2.51321	0.96990	-0.58930
O	2.13294	1.99077	-1.13324
H	4.45825	1.63529	-0.50343
N	-3.04220	1.66268	0.43263
C	-3.84090	0.59011	0.12470
H	-4.90839	0.78151	0.15094
C	-3.33293	-0.62081	-0.18181
C	-4.16659	-1.82169	-0.51055
H	-5.23183	-1.57764	-0.48039
H	-3.92281	-2.20222	-1.50670
H	-3.97320	-2.63368	0.19659
C	-1.87728	-0.76273	-0.17902
O	-1.31123	-1.83618	-0.39903
N	-1.14570	0.37871	0.09105
H	-0.11402	0.31048	-0.01628
C	-1.65437	1.61187	0.45090
O	-0.97816	2.56961	0.76409
H	-3.43916	2.55450	0.68521

TG

N	-1.86564	2.03529	-0.62345
C	-3.05901	2.73022	-0.54545
H	-3.11563	3.78437	-0.77566
N	-4.05413	1.97932	-0.17271
C	-3.50060	0.72767	-0.00328
C	-4.11033	-0.51750	0.38031
O	-5.25760	-0.78583	0.67294
N	-3.13064	-1.55652	0.38312
H	-3.50709	-2.45835	0.64489
C	-1.79755	-1.43547	0.09807
N	-1.03064	-2.54005	0.18870
H	-1.45276	-3.44423	0.31395
H	-0.07084	-2.50606	-0.16845
N	-1.24894	-0.27964	-0.23258
C	-2.14352	0.74298	-0.28011

H	-0.98021	2.38876	-0.94859
N	1.63267	-0.12048	-0.04392
C	2.30283	1.02413	0.32087
H	1.66312	1.86061	0.58345
C	3.64793	1.11372	0.36545
C	4.39771	2.34760	0.76479
H	3.71077	3.16116	1.01155
H	5.03259	2.14956	1.63317
H	5.05802	2.67669	-0.04275
C	4.42340	-0.07505	0.00330
O	5.63969	-0.14495	-0.00991
N	3.65044	-1.19257	-0.34189
H	4.15681	-2.03590	-0.58094
C	2.27921	-1.27905	-0.37264
O	1.69207	-2.31826	-0.67001
H	0.59986	-0.13700	-0.10342

TT

N	4.04942	-1.13750	-0.00140
C	4.31028	0.21168	-0.00118
H	5.36081	0.48161	-0.00210
C	3.32874	1.13584	0.00009
C	3.56537	2.61533	0.00015
H	4.63518	2.83978	-0.00013
H	3.10836	3.08030	-0.87804
H	3.10879	3.08017	0.87861
C	1.94797	0.65413	0.00112
O	0.97647	1.41002	0.00204
N	1.77510	-0.71907	0.00114
H	0.80136	-1.06108	0.00144
C	2.77169	-1.67477	-0.00042
O	2.56999	-2.87308	-0.00063
H	4.79762	-1.81358	-0.00220
N	-4.04966	1.13724	-0.00094
C	-4.31023	-0.21198	-0.00068
H	-5.36070	-0.48217	-0.00112
C	-3.32849	-1.13594	0.00007
C	-3.56482	-2.61547	0.00006
H	-4.63458	-2.84011	0.00176
H	-3.10941	-3.08008	-0.87916
H	-3.10645	-3.08051	0.87749
C	-1.94786	-0.65396	0.00064
O	-0.97622	-1.40966	0.00152
N	-1.77522	0.71930	0.00016
H	-0.80156	1.06151	0.00043

C	-2.77201	1.67478	-0.00060
O	-2.57060	2.87313	-0.00090
H	-4.79797	1.81318	-0.00145

TU

N	-3.94030	-0.89924	-0.00324
C	-4.05117	0.47015	-0.00247
H	-5.06558	0.85414	-0.00421
C	-2.97386	1.28087	-0.00002
C	-3.04678	2.77741	0.00061
H	-4.08559	3.11761	-0.00145
H	-2.54394	3.18917	0.88031
H	-2.54013	3.19010	-0.87646
C	-1.65481	0.65026	0.00187
O	-0.60598	1.29512	0.00369
N	-1.63355	-0.73352	0.00164
H	-0.70372	-1.18072	0.00286
C	-2.72933	-1.57412	-0.00107
O	-2.66092	-2.78704	-0.00161
H	-4.75821	-1.48911	-0.00438
N	4.36315	0.49641	-0.00282
C	4.48418	-0.86771	-0.00255
H	5.49863	-1.24913	-0.00446
C	3.40245	-1.66993	-0.00005
H	3.48162	-2.74729	0.00018
C	2.07649	-1.07239	0.00249
O	1.03252	-1.72096	0.00479
N	2.04956	0.31538	0.00190
H	1.11711	0.75907	0.00303
C	3.14010	1.16049	-0.00102
O	3.06820	2.37238	-0.00179
H	5.17373	1.09665	-0.00510

UU1

N	-4.14001	0.95610	-0.00084
C	-2.95462	1.65307	-0.00024
H	-3.05015	2.73241	-0.00055
C	-1.76020	1.03228	0.00067
C	-1.72332	-0.41952	0.00101
O	-0.71214	-1.11286	0.00183
N	-2.97379	-1.03788	0.00033
H	-2.98212	-2.05044	0.00053
C	-4.21362	-0.42603	-0.00059
O	-5.26524	-1.03416	-0.00116

H	-5.02881	1.43307	-0.00152
H	-0.81606	1.56589	0.00112
O	1.30682	1.92473	0.00097
C	2.24822	1.14117	0.00032
N	2.03078	-0.23527	0.00038
H	1.05273	-0.55869	0.00101
O	2.75439	-2.41422	-0.00012
C	2.99326	-1.22357	-0.00020
N	4.29744	-0.73783	-0.00090
H	5.01540	-1.44606	-0.00133
H	5.66654	0.83148	-0.00152
C	4.60848	0.59643	-0.00097
C	3.64995	1.54150	-0.00038
H	3.88193	2.59670	-0.00040

UU2

N	-4.13730	-0.96119	0.00084
C	-2.95019	-1.65466	0.00048
H	-3.04286	-2.73426	0.00079
C	-1.75763	-1.03026	-0.00021
C	-1.72428	0.42204	-0.00058
O	-0.71542	1.11773	-0.00115
N	-2.97692	1.03647	-0.00014
H	-2.98791	2.04907	-0.00034
C	-4.21487	0.42113	0.00054
O	-5.26845	1.02551	0.00094
H	-5.02483	-1.44054	0.00137
H	-0.81283	-1.56165	-0.00047
O	2.75137	2.45449	-0.00035
C	3.04493	1.27415	-0.00040
N	2.04688	0.28624	-0.00052
H	1.06882	0.60784	-0.00082
O	1.33282	-1.89538	-0.00033
C	2.23677	-1.07130	-0.00034
N	3.56516	-1.46423	0.00023
H	3.71772	-2.46082	0.00047
H	5.60323	-1.02334	0.00099
C	4.61552	-0.57746	0.00052
C	4.41042	0.75067	0.00028
H	5.22738	1.45796	0.00056

UU3

N	-4.16826	1.17658	0.00018
C	-2.85304	1.57875	0.00024

H	-2.69359	2.65052	0.00040
C	-1.83439	0.69885	0.00012
C	-2.13027	-0.72584	-0.00009
O	-1.30725	-1.63074	-0.00017
N	-3.49591	-1.03261	-0.00013
H	-3.74127	-2.01463	-0.00024
C	-4.55943	-0.15078	0.00000
O	-5.72414	-0.49862	-0.00000
H	-0.79965	1.02538	0.00019
O	1.30725	1.63075	-0.00010
C	2.13027	0.72585	-0.00009
N	3.49592	1.03260	-0.00006
H	3.74127	2.01463	-0.00004
O	5.72415	0.49861	0.00008
C	4.55943	0.15078	0.00002
N	4.16826	-1.17658	0.00002
H	2.69358	-2.65052	0.00002
C	2.85303	-1.57875	-0.00001
C	1.83439	-0.69885	-0.00006
H	0.79965	-1.02538	-0.00006
H	4.92196	-1.84683	0.00009
H	-4.92196	1.84682	0.00031
